# Effects of Phosphorus Doping on Amorphous Boron Nitride’s Chemical, Sorptive, Optoelectronic, and Photocatalytic Properties

**DOI:** 10.1021/acs.jpcc.4c02314

**Published:** 2024-07-24

**Authors:** Ioanna Itskou, Andreas Kafizas, Irena Nevjestic, Soranyel Gonzalez Carrero, David C. Grinter, Hassan Azzan, Gwilherm Kerherve, Santosh Kumar, Tian Tian, Pilar Ferrer, Georg Held, Sandrine Heutz, Camille Petit

**Affiliations:** † Barrer Centre, Department of Chemical Engineering, Imperial College London, London SW7 2AZ, U.K.; ‡ Department of Chemistry, Molecular Sciences Research Hub, Imperial College London, London W12 7TA, U.K.; § London Centre for Nanotechnology, Imperial College London, London SW7 2AZ, U.K.; ∥ Department of Materials, Imperial College London, London SW7 2AZ, U.K.; ⊥ Department of Chemistry, Centre for Processable Electronics, Imperial College London, London W12 7TA, U.K.; # Diamond Light Source, Harwell Science and Innovation Campus, Didcot OX11 0DE, U.K.

## Abstract

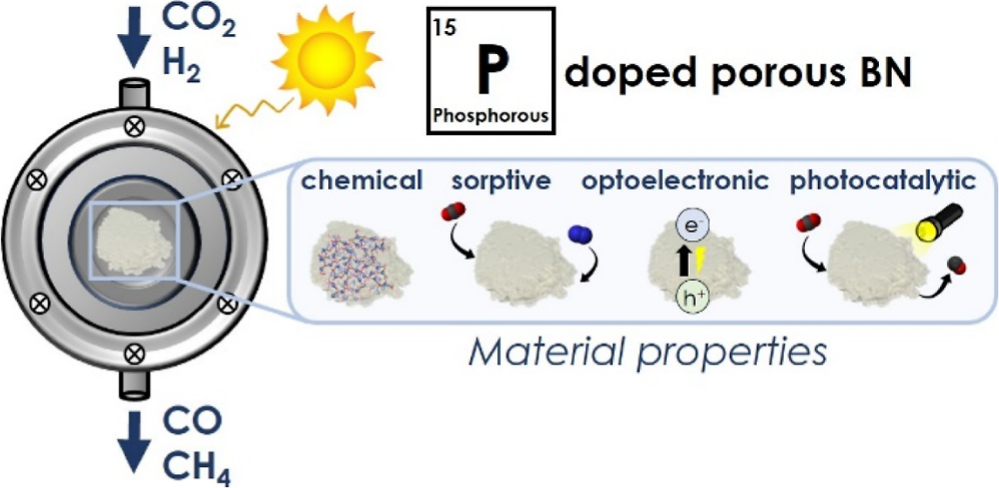

Amorphous porous boron nitride (BN) represents a versatile material platform with potential applications in adsorptive molecular separations and gas storage, as well as heterogeneous and photo-catalysis. Chemical doping can help tailor BN’s sorptive, optoelectronic, and catalytic properties, eventually boosting its application performance. Phosphorus (P) represents an attractive dopant for amorphous BN as its electronic structure would allow the element to be incorporated into BN’s structure, thereby impacting its adsorptive, optoelectronic, and catalytic activity properties, as a few studies suggest. Yet, a fundamental understanding is missing around the chemical environment(s) of P in P-doped BN, the effect of P-doping on the material features, and how doping varies with the synthesis route. Such a knowledge gap impedes the rational design of P-doped porous BN. Herein, we detail a strategy for the successful doping of P in BN (P-BN) using two different sources: phosphoric acid and an ionic liquid. We characterized the samples using analytical and spectroscopic tools and tested them for CO_2_ adsorption and photoreduction. Overall, we show that P forms P–N bonds in BN akin to those in phosphazene. P-doping introduces further chemical/structural defects in BN’s structure, and hence more/more populated midgap states. The selection of P source affects the chemical, adsorptive, and optoelectronic properties, with phosphoric acid being the best option as it reacts more easily with the other precursors and does not contain C, hence leading to fewer reactions and C impurities. P-doping increases the ultramicropore volume and therefore CO_2_ uptake. It significantly shifts the optical absorption of BN into the visible and increases the charge carrier lifetimes. However, to ensure that these charges remain reactive toward CO_2_ photoreduction, additional materials modification strategies should be explored in future work. These strategies could include the use of surface cocatalysts that can decrease the kinetic barriers to driving this chemistry.

## 1 Introduction

There has been a particular spotlight recently on the forms of boron nitride (BN) materials, in which B and N atoms are alternatingly connected to each other with single and double bonds to form six-member rings. Such forms include hexagonal BN (hBN) and its turbostratic and amorphous derivatives. While hBN has been long used industrially as a lubricant in the cosmetic, coating, and painting industries, it has found new potential applications in thermal management,^1^ catalysis, and adsorption/molecular separations.^2^

The structural differences that come with moving from crystalline hBN to turbostratic or amorphous BN have partly driven this opening of new applications. For instance, in its amorphous form, BN becomes porous and can therefore be used in gas separations and storage applications, e.g., CO_2_ capture and water purification.^2^ Another example is that while hBN is an insulator and exhibits a wide band gap, amorphous BN can be tuned to become a semiconductor and harvests both visible and UV light.^3^ Porosity and optoelectronic properties, such as band gap and charge carrier behavior, can regulate photocatalytic properties. Hence, we observe studies on the use of amorphous and porous BN as photocatalyst, e.g., for water treatment,^4,5^ H_2_ production,^4^ or CO_2_ photoreduction.^3,6−8^

Beyond the structural changes that they bring, the turbostratic and amorphous forms of BN open a route for chemical tuning owing to their defective nature. Hence, they provide a new dimension to tailoring and improving the performance of a material for a given application. A common pathway to tuning the chemistry of a material is doping with metal or nonmetal heteroatoms. By introducing different elements to the material, one can change its chemical, thermal, sorptive, optoelectronic, and catalytic properties. The nonmetal dopants employed so far for BN materials include: C, O, S, F, P, Cl, and Si.^2^ We note for instance that C-doping can enhance CO_2_ adsorption of hBN^9−11^ as well as sulfur removal from fuels.^12^ Often, the creation of ultramicropores caused by doping explains the enhanced CO_2_ uptake. Research groups have also improved BN materials’ light absorption and photocatalytic performance by introducing heteroatoms which altered the band gap and electron transfer mechanisms.^2,13^ More specifically, studies have suggested that C-doping of BN (5–10 wt %) results in the replacement of B and N atoms with C in basal planes, causing a reduction of the band gap (from 5.5 to 1.0–2.0 eV) and enhanced gas (mostly CO_2_) adsorption and product (CO or H_2_) evolution rates.^8,14,15^ Similarly, doping with O (5–10 at. %) results in reduced band gap (to 1.5–2.9 eV), altered magnetic properties, and enhanced CO_2_ adsorption and CO production.^3,16−19^ Si-doping (4–5 at. %) within basal planes of BN nanotubes reduced the band gap from 1.9 to 0.3 eV.^20^ Some studies have also reported that doping BN with F (up to 1 at. %) and S (5 at. %) within basal planes enhances the CO evolution and H_2_ production rates, respectively, during photocatalysis.^21,22^ Overall, the studies observe that doping extends the light harvesting (reduced band gap). Many studies also show an improved photocatalytic performance for different reactions and attribute this enhancement to extended light harvesting and/or improved charge carrier lifetimes and transport. Direct confirmation of the latter is not always reported.

Phosphorus (P) has been used to dope activated carbon to enhance its adsorptive properties, and graphitic carbon nitride (g-C_3_N_4_) has been used to change its optoelectronic properties and improve its photocatalytic activity. Activated carbon is a common adsorbent and bears resemblance with amorphous BN in terms of porosity, while g-C_3_N_4_ is a promising photocatalyst and has a very similar structure to BN. These studies place P as a promising dopant to be explored for amorphous BN.^23−28^ P-doping on BN using phosphorus as the P-precursor has been experimentally studied for the adsorption of heavy metals from flue gas.^29^ It was found that P-doped BN adsorbs zinc (primarily) and copper (secondarily) more strongly compared to other heavy metals. Another study reported P-doped BN nanosheets supported on graphene, as an interlayer for Li–S batteries, and showed increased cyclic stability and rate capability due to synergy between graphene physical barrier and P-BN chemical adsorption. P-doped BN has also been reported as an efficient catalyst for the dehydrogenation of ethylbenzene, while the introduction of B defects to the structure enhances its catalytic activity.^30−32^ Moreover, P-doped BN nanosheets have previously been incorporated in a Z-scheme heterojunction with ZnIn_2_S_4_ for photocatalytic H_2_ and H_2_O_2_ evolution.^33^ The material showed enhanced H_2_ and H_2_O_2_ production rates and good stability, potentially due to the promotion of charge separation and inhibition of their recombination. Theoretical studies have also investigated the mechanical and electronic properties and thermodynamic stability of P-doped hBN layers, where P would replace either B or N atoms in the structure.^34−39^ P incorporation would cause bond lengthening and structural distortion due to inclusion of their sp^3^ bonds in the otherwise sp^2^ bonding system of h-BN. Especially P_B_ defects could affect the optical properties of BN and reduce the band gap promoting visible light harvesting.

Together, these studies point to the potential of P-doping to tune the chemical, sorptive, and optoelectronic properties of amorphous BN. Yet, they also highlight a lack of a fundamental understanding and direct experimental evidence of the effect of such doping. For instance, there is no direct experimental confirmation of how P atoms incorporate into amorphous BN, how this is influenced by the P-containing precursor, and how P chemical environment might impact BN’s properties. Questions regarding the reason(s) behind the increased sorptive properties of P-doped BN remain. In addition, the mechanism(s) by which P-doping changes the charge carrier behavior are yet to be elucidated. Furthermore, isolating the effect of P-doping becomes particularly difficult when BN is used in conjunction with other materials to form composites. Shining light on these questions could lead to the rational design of BN-based materials with improved performance for targeted applications.

Herein, we aim to address the above knowledge gap and specifically investigate: (i) how P incorporates into the structure of porous amorphous BN, and (ii) how this doping affects the chemical, sorptive, optoelectronic, and photocatalytic properties of the material. Toward this goal, we doped porous amorphous BN with P using two different P-containing precursors: phosphoric acid and ionic liquid. For the first time, we carried out an in-depth advanced characterization of P-doped BN (P-BN) materials’ chemical, sorptive, optoelectronic, and photocatalytic properties, and compared them with as-prepared pristine BN. We used (i) Fourier transform infrared spectroscopy (FTIR), X-ray photoelectron spectroscopy (XPS), and near edge X-ray absorption fine structure (NEXAFS) to measure the chemical, (ii) X-ray diffraction (XRD) and N_2_ sorption (−196 °C) to measure the structural, (iii) CO_2_ sorption (15, 25, 35 °C) to measure the sorptive, and (iv) ultraviolet–visible diffuse reflectance spectroscopy (DRS-UV/vis), steady state photoluminescence (PL), time-correlated single photon counting (TCSPC), transient absorption spectroscopy (TAS), and electron paramagnetic resonance (EPR) to measure the optoelectronic and charge carrier properties. Finally, we tested the materials for CO_2_ photoreduction in the gas phase using a photocatalytic setup equipped with a solar simulator and carried out control tests.

## 2 Methods

### 2.1 Synthesis

Overall, we synthesized all BN samples using a two-step approach as described below, and for P-doped porous BN, we employed two different P-containing dopants: an ionic liquid and phosphoric acid.

#### 2.1.1 Synthesis of Pristine BN

Boric acid (3.71 g, >99.5%, ACS reagent, Sigma-Aldrich) and melamine (3.78 g, 99.0%, Sigma-Aldrich) were mixed and dissolved in a beaker containing 50 mL of deionized water overnight at 80 °C using magnetic stirring. Once all the water had evaporated, the mixture was transferred to a drying oven at 65 °C for 24 h and then heated in a vacuum oven at 110 °C overnight. Approximately 2 g of the obtained powder were ground, loaded inside an alumina crucible, and placed into a horizontal tubular furnace. Initially the mixture was purged under nitrogen (N_2_) gas (zero grade, 99.998%, BOC) at 250 cm^3^ min^–1^ for 2 h to remove as much air and moisture as possible. Then, the furnace was heated up to 1050 °C at a heating rate of 10 °C min^–1^ under N_2_ flow (50 cm^3^ min^–1^) and the temperature was held for 3.5 h. After that, the furnace was allowed to cool naturally to room temperature under the same N_2_ flow. The obtained porous BN powder was manually ground in an agate mortar and labeled as BN.

#### 2.1.2 Synthesis of P-Doped BN Using Ionic Liquid as P-Dopant Source

Boric acid (3.71 g, >99.5%, ACS reagent, Sigma-Aldrich), melamine (3.78 g, 99.0%, Sigma-Aldrich), and 1-butyl-3-methylimidazolium hexafluorophosphate (BMIM-PF_6_, 2 mL, >98.0%, Acros Organics) were used as precursors and the synthesis was carried out in the same way as for pristine BN. This time, the furnace was heated up under different N_2_ flows (50 or 100 cm^3^ min^–1^) and once the final temperature was reached, it was allowed to cool down naturally under the same N_2_ flow. The products were manually ground in an agate mortar and labeled P-BN_IL-HF_ for 100 cm^3^ min^–1^ flow, and P-BN_IL-LF_ for 50 cm^3^ min^–1^ flow.

#### 2.1.3 Synthesis of P-Doped BN Using Phosphoric Acid as P-Dopant Source

The same process as aforementioned was followed, with the difference that phosphoric acid (0.95 g, >98.0%, Acros Organics) was added instead of the ionic liquid. The products were manually ground in an agate mortar and labeled P-BN_PA-HF_ for 100 cm^3^ min^–1^ flow, and P-BN_PA-LF_ for 50 cm^3^ min^–1^ flow.

### 2.2 Methods

#### 2.2.1 Characterization of Chemical Properties

For FTIR measurements, a Cary 630 FTIR spectrometer (Agilent) equipped with an attenuated total reflectance (ATR) accessory was used. Samples were manually ground in an agate mortar, and the spectra were recorded after 32 repetitions per sample in the 650–4000 cm^–1^ range, with a 2 cm^–1^ resolution.

For the XPS measurements, a high-throughput K-Alpha X-ray Photoelectron Spectrometer (Thermo Scientific) equipped with a monochromatic Al Kα source (*hv* = 1486.6 eV) was used. Samples were manually ground in an agate mortar and mounted on the XPS holder by using conductive carbon tape. The X-ray power gun was set to 72 W. Data analysis on the B 1s, N 1s, C 1s, and P 2p spectra was performed using the Thermo Avantage software. The adventitious carbon (C–C) peak set at 284.8 eV was used for binding energy calibration.

NEXAFS spectroscopy experiments were performed at the VerSoX B07-B beamline at Diamond Light Source, UK.^40,41^ Samples were manually ground in an agate mortar and mounted on a multisample NEXAFS holder (Omicron plate) using copper-supported conductive carbon tape. The photon energy resolutions were ∼80 meV (B, N, and O K-edges) and ∼500 meV (P K-edge). The spectra were recorded in total electron yield (TEY) mode at room temperature under 1 mbar of He to compensate for sample charging.

#### 2.2.2 Characterization of Structural Properties

Powder XRD data were collected using an X’Pert Pro X-ray diffractometer (PANalytical) in the reflection mode, with an anode voltage of 40 kV and an emission current of 20 mA using a monochromatic Cu Kα radiation (λ = 1.54178 Å). Samples were manually ground in an agate mortar and deposited on a solid holder. The XRD detector was a silicon strip detector X’Celerator.

N_2_ (−196 °C) sorption isotherms were obtained using a 3Flex Sorption Analyzer (Micromeritics). Prior to the measurements, the samples were degassed ex situ using a VacPrep Degasser (Micromeritics) at 140 °C overnight at 0.02 mbar. Then, they were in situ degassed at 120 °C for 4 h down to 7 × 10^–5^ bar using the 3Flex Sorption Analyzer. The specific surface areas were calculated using the Brunauer–Emmett–Teller (BET) method following the BETSI approach, which expands on the Rouquerol criteria to produce a standard BET area assignment.^42−44^ The total pore volume was estimated from the amount of adsorbed N_2_ at *P*/*P*_0_ = 0.97. The micropore volume was determined using the Dubinin–Radushkevich method.^45^ The ultramicropore volume was determined using the Horvath–Kawazoe method.^46^

#### 2.2.3 Characterization of Optoelectronic Properties

For the DRS-UV/vis measurements, a UV/vis-IRS-2600Plus (Shimadzu) spectrophotometer was used, equipped with an integrated sphere attachment. The spectral bandwidth was set to 2 nm, with BaSO_4_ as a reference. The samples were loaded and pressed into the holder until they covered the entire surface. Absorbance spectra were derived using the Kubelka–Munk function.^47,48^

PL spectra were recorded at room temperature by using a Cary Eclipse fluorescence spectrometer (Agilent). For the measurements, the excitation wavelength was set initially to 200 nm and later to 282 nm, with 5 nm excitation/emission slits, 0.5 s dwell time, 1 nm data interval, and 800 V photomultiplier tube (PMT) voltage.

TCSPC measurements were carried out by using a commercial TCSPC setup (HORIBA DeltaFlex) equipped with a pulsed LED excitation source (HORIBA NanoLED series) and a fast rise-time photomultiplier detector (HORIBA PPD-650 and PPD-900). The instrument response function (IRF) was measured at the wavelength of the excitation source (282 nm). During measurements, a suitable long pass filter was inserted between the sample and detector to block off scattered excitation light.

TAS was recorded using a home-built setup in diffuse reflection mode. A Nd:YAG laser (OPOTEK Opolette 355 II, 7 ns pulse width) was used to generate 355 nm excitation pulses of 100 μJ cm^−2^. A broadband probe light was generated from a quartz halogen lamp (Bentham IL1) and long pass filters were placed before the samples to reduce short wavelength irradiation of the sample. The light was collected in diffuse reflectance mode by a 2 in. diameter, 2 in. focal length lens, and relayed to a monochromator to select the probe wavelength. A long pass filter was positioned at the entrance of the monochromator to block the scattered laser light. The collected light was focused onto a Si photodiode detector (Hamamatsu S3071). Sub-ms data were processed by an electronic amplifier (Costronics) and recorded on an oscilloscope. Data on the ms time scale were simultaneously recorded by a DAQ card (National Instruments). Acquisitions were triggered by scattering from the laser excitation measured by a photodiode (Thorlabs DET10 A). A minimum of 200 laser pulses were averaged together and processed using LabVIEW home-built software. The measurements were performed on powdered samples in air.

EPR measurements were performed using an Elexsys E500T continuous-way CW EPR spectrometer (Bruker) operating at X-band frequencies (9.5–9.9 GHz/0.35 T), and equipped with an ER4118-X MD5 resonator (Bruker) and cryogen free variable temperature cryostat for EPR (Cryogenic). The microwave frequencies used were 9.61 GHz with a microwave power of 2 mW, 100 kHz modulation frequency with 2G modulation amplitude at room temperature, and 10 kHz modulation frequency, 2G modulation amplitude, and 8 μW power at −268 °C. Irradiation measurements of the samples were carried out under direct illumination of a 300 W Xe arc lamp through an optical access port. The samples were placed inside 4 mm EPR quartz tubes, and spectra were recorded at (i) room temperature in air and (ii) −268 °C in He. Each EPR tube is filled with the sample to the same height. EPR spectra are normalized for the mass of the samples. Care was taken to position the center of each sample in the center of the resonator. Simulations of EPR spectra were performed by using the EasySpin toolbox (version 6.0.2) for MATLAB.^49^

#### 2.2.4 CO_2_ Adsorption

CO_2_ adsorption isotherms were measured right after finishing with N_2_ sorption measurements used for porosity analyses. The samples were degassed in situ at 120 °C for 4 h down to 7 × 10^–5^ bar using the same 3Flex Sorption Analyzer. CO_2_ gas (research grade, 99.999%, BOC) was used, and the sorption isotherms were measured sequentially at 15, 25, and 35 °C up to 1 bar. The isotherms were fitted using dual site Langmuir model^50^
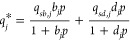
1
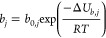
2
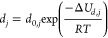
3where *q*_j_* is the adsorbed amount of gas *j* at pressure *p* and temperature *T*, *b* and *d* are adsorption coefficients, *b*_0_, *d*_0_, Δ*U*_*b*_, and Δ*U*_*d*_ are constants, *R* is the universal gas constant, and *q*_*sb*_ and *q*_*sd*_ are saturation capacities.

Afterward, the isosteric heat of adsorption was calculated for each material using the virial fit on our experimental data at the three temperatures. The virial equation is^51−53^
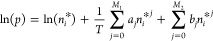
4where *n*_i_* is the amount of CO_2_ adsorbed at pressure *p* and temperature *T*, and *a* and *b* are characteristic virial coefficients. In this study, we used *M*_1_ = 3 and *M*_2_ = 1 to provide a good fit. The Langmuir and virial isotherm fittings were carried out with MATLAB R2020a (The Mathworks Inc.) using the in-house software package isothermFittingTool.^54^

#### 2.2.5 CO_2_ Photoreduction

CO_2_ photoreduction experiments were performed in a homemade setup using a stainless steel photoreactor (17.7 cm^3^) equipped with a fused quartz window for irradiation. Approximately 20 mg of sample was ground and dry-cast onto a stainless-steel holder (7.1 cm^2^) and activated at 110 °C in a vacuum oven overnight. Then, the sample holder was placed inside the photoreactor and vacuumed at approximately 0.013 bar for 15 min. CO_2_ gas (80 cm^3^ STP min^–1^, research grade, 99.999%, BOC) and H_2_ (40 cm^3^ STP min^–1^, produced in a H_2_ generator) were mixed and flown for 15 min. Finally, the photoreactor was filled with approximately 2 bar of the gas mixture and sealed. The sample was then irradiated for 5 h with a solar simulator equipped with a 100 W Xe arc lamp (Oriel LCS-100 solar simulator, Newport) at a distance of 15 cm from the sample, providing the intensity at the catalyst surface to be close to 1 sun power (80 mW cm^–2^). A long pass UV filter (λ < 400 nm, LOT Quantum Design) was used to conduct the test under visible light irradiation. The gaseous products were detected and analyzed using a GCMS-QP2020 NX gas chromatographer (Shimadzu) equipped with Rt-Q-Bond Plot column (30 m, 0.32 mm ID, 10 μm, Shimadzu) and molecular sieve SH-Msieve 5A plot column (30 m, 0.32 mm ID, 30 μm, Shimadzu) in series. The tests were repeated three times on fresh samples. Control experiments were performed: (a) without irradiation, (b) with the stainless-steel holder and no sample, (c) under a N_2_/H_2_ atmosphere, and (d) under an isotopic ^13^CO_2_/H_2_ atmosphere (99 at. % ^13^CO_2_, BOC).

## 3 Results and Discussion

**Figure 1:**
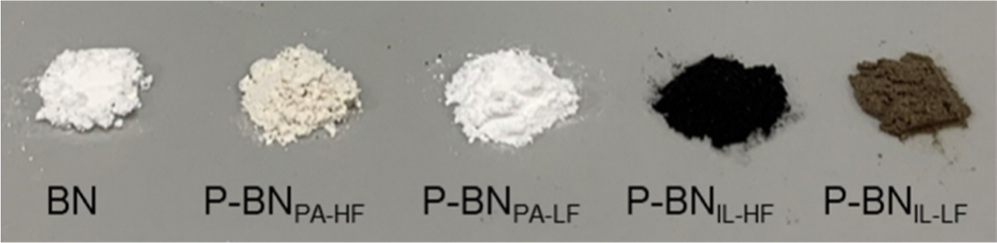
Optical images of the as-prepared pristine BN and P-BN samples.

After completing the three synthesis routes described in Section 2.1, we obtained a pristine boron nitride (BN) sample and four P-doped boron nitride (P-BN) samples: P-BN_IL-HF_ and P-BN_IL-LF_ using ionic liquid as the P-containing source, and P-BN_PA-HF_ and P-BN_PA-LF_ using phosphoric acid. Optical images of the synthesized samples are shown in Figure 1. Pristine BN sample is white, while P-BN_PA_ samples show a yellowish tone, especially P-BN_PA-HF_ which was produced using a high N_2_ flow during synthesis. On the other hand, P-BN_IL_ samples exhibit a dark color, with P-BN_IL-HF_ being dark brown and P-BN_IL-LF_ a lighter brown. The changes in color originate from changes in the band structure of the materials, which we discuss in more detail in Section 3.3. In general, samples produced using a lower N_2_ flow during synthesis seemed to exhibit a lighter color, which is an aspect we comment on further below.

### 3.1 Physical and Chemical Properties

**Figure 2:**
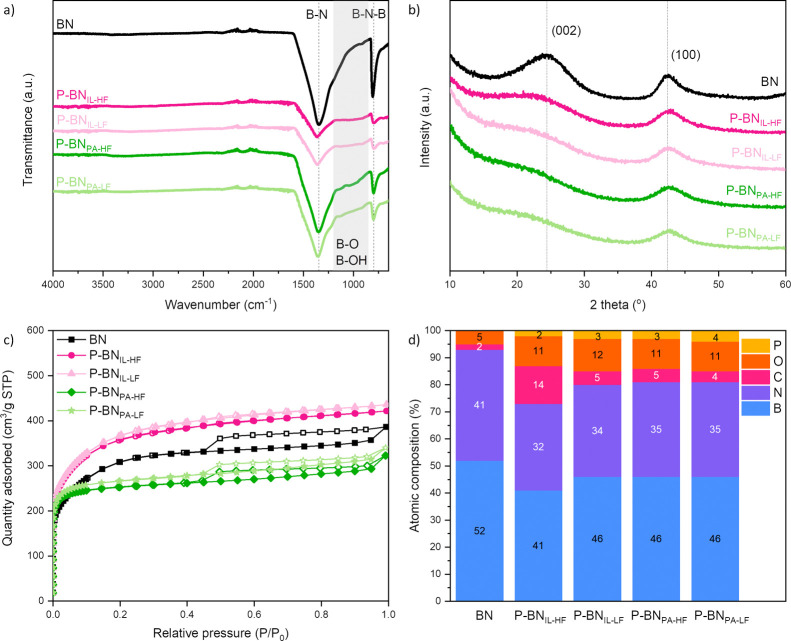
(a) ATR–FTIR spectra with highlighted IR bands, (b) XRD data with highlighted plane peaks, (c) N_2_ sorption (−196 °C) isotherms, and (d) atomic composition based on XPS analysis of pristine BN and P-doped BN samples.

Following the synthesis of the materials, we examined their physical and chemical properties using a wide range of characterization techniques, which are summarized in Table 1. We analyzed the functional groups in pristine BN and P-BN samples using ATR–FTIR (Figure 2a). All samples show the typical bands at ∼1350 and at ∼800 cm^–1^ corresponding to B–N stretching and B–N–B bending, respectively.^55^ Less intense bands appearing in the 850–1200 cm^–1^ range—especially in P-BN samples—suggest the existence of B–O and B–O–H bonds.^56^ This agrees with previous studies on porous BN, showing the replacement of planar N atoms with O, and also the creation of –OH functional groups on the edges of the BN structure.^2,19,57,58^ We then analyzed our samples using XRD and present the results in Figure 2b. They confirm the formation of hexagonal BN [*P*6_3_/*mnc*; *a* = *b* = 2.50399(5) Å, *c* = 6.6612(5) Å; α = β = 90.0°, γ = 120.0°].^59^ The large breadth of the observed peaks shows that the materials produced contain amorphous and highly nanocrystalline regions, where we estimate using the Scherrer relation that the crystals formed are on average in the region of 2 nm in size.^60^ There is no apparent shift of the (002) or (100) plane peaks from pristine BN to the doped samples, meaning that the *d*-spacing and periodicity with respect to the crystalline hexagonal phase of BN did not change with doping. However, the primary (002) peak is reduced in intensity and broader upon P-doping, indicating that the average crystal size decreases and there is a greater preference for P-doped samples to grow in the (100) crystal plane. We examined the porosity and surface area of the materials using N_2_ sorption at −196 °C, and present the N_2_ sorption isotherms in Figure 2c and the textural parameters in Table S1. Pristine BN (BET area: 1135 m^2^ g^–1^) and P-BN_PA_ samples (BET area: 989–1036 m^2^ g^–1^) show type I/IV sorption isotherms with H3/H4 hysteresis loops, indicative of meso- and microporosity and narrow slit-like pores, respectively. We observe a clear difference when moving to P-BN_IL_ samples (BET area: 1321–1351 m^2^ g^–1^) that show type I isotherms with no hysteresis loop, indicative of pure microporosity. Overall, all of our samples are more porous than reported in the literature for doped P-BN. The type I isotherm of the P-BN_IL_ is quite unique for P-BN samples compared to the previous studies.

**Table 1:** Overview of the Chemical, Physical, and Sorptive Properties Studied and the Related Techniques Employed

properties	techniques
elemental composition	XPS
chemical environment and bonding type	FTIR, XPS, NEXAFS, EPR
crystallinity	XRD
porosity	volumetric gas sorption analyzer, N_2_ sorption at –196 °C
CO_2_ adsorption capacity	volumetric gas sorption analyzer, CO_2_ sorption at 15, 25, and 35 °C

From a chemical analysis point of view, we assessed the atomic composition of the materials using XPS, and show the results in Figure 2d. The P-doping level follows the trend: P-BN_IL-HF_ (2 at. %) < P-BN_IL-LF_ = P-BN_PA-HF_ (3 at. %) < P-BN_PA-LF_ (4 at. %). This content level is slightly higher than that reported in the experimental literature for P-BN samples. Using phosphoric acid as P source generally results in higher P at. % than when using ionic liquid. Lower N_2_ flow rate during the synthesis (i.e., higher residence time) leads to higher doping amount, as gaseous products linger and react with the material for longer times than in higher flow. The difference in residence time could explain the difference in color, mainly between P-BN_IL-HF_ and P-BN_IL-LF_. The B/N ratio (∼1.3) is similar in all samples, while O and C contents are higher in P-BN (∼11 and 5 at. %, respectively) than in pristine BN (5 and 2 at. %). The C content is significantly higher in P-BN_IL-HF_ (14 at. %)—the optically darkest sample. C impurities are caused by the replacement of B and N atoms (creating C–C, C–B, and C–N bonds), while O usually replaces N within the structure, and also creates –OH groups on the edges (forming B–O, C–O, and O–H bonds).^2,61,62^ Although amorphous pristine BN is inherently defective (C and O presence, N vacancies), we can conclude that P-doping introduces additional defects into the BN structure compared to pristine BN.

**Figure 3:**
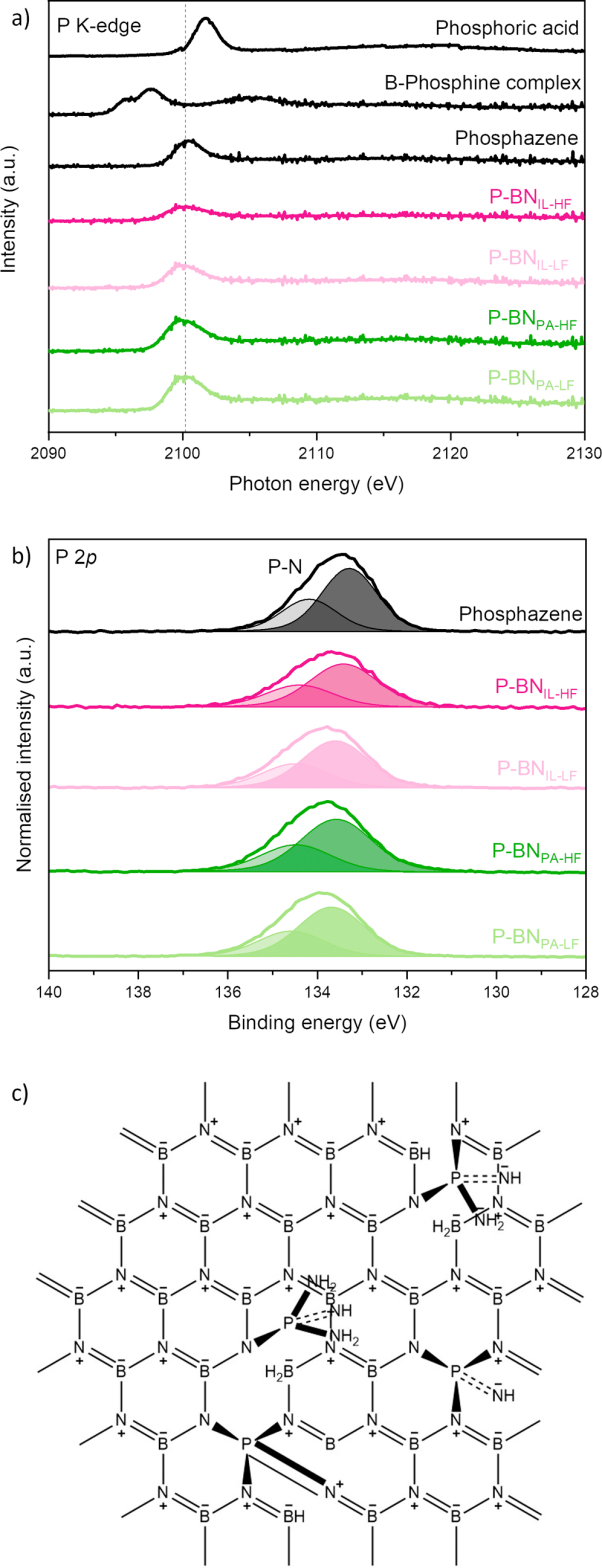
(a) P K-edge NEXAFS spectra of the three P-containing reference samples and the P-doped BN samples, (b) P 2p XPS deconvolution spectra of phosphazene reference sample and P-doped BN samples, and (c) proposed chemical structure of the chemical environment around P atoms in P-doped BN samples, as suggested by NEXAFS and XPS analyses. We note that in this proposed structure, we focus on how P atoms may be embedded, and have not included O or C presence discussed in the text.

Having confirmed the successful P-doping of our materials, we further investigated for the first time their chemical structure and environment around P atoms using NEXAFS. For this purpose, we used three reference samples, all containing different P bonds (the details and structures of the reference samples are shown in Figure S1): (i) phosphoric acid containing P–O and P=O bonds, (ii) B–phosphine complex containing P–B and P–C bonds, and (iii) phosphazene containing P–N and P=N bonds. The results of our measurements are presented in Figure 3a. All P-BN samples show the same main P K-edge peak at ∼2100 eV as that of the phosphazene reference sample. We can conclude that, regardless of the P source, the P-doped samples share the same P-containing functionalities, and these are akin to the ones of the phosphazene reference sample. Hence, these analyses suggest the creation of P–N or P=N bonds in the BN structure. There are no peaks corresponding to the same energy level with the other two reference samples; hence, we conclude that P atoms mainly form bonds with N, rather than with C, B, or O. B K-edge, N K-edge, and O K-edge NEXAFS spectra of pristine BN and P-BN samples can be found in Figure S2. The chemical environment around N and O atoms appears similar between pristine and P-doped BN, yet P-BN shows more π_BN_2_O_ and π_BO_3__ bonds than π_BNO_2__ (see Table 2).^17,63^ Therefore, it is more likely that O replaces either one or three N atoms surrounding B in P-doped BN. Overall, NEXAFS provides strong, if not definite, support for the creation of P–N or P=N bonds. This finding is in accordance with some XPS studies reported before, though other researchers have tentatively assigned XPS bands to P–O and P–B bonds.

**Table 2:** π_BN_3__/π_BN_2_O_/π_BNO_2__/π_BO_3__ Ratio in Pristine and P-Doped BN Samples, as Derived from B K-Edge NEXAFS Spectra (Figure S2)

sample	π_BN_3__	π_BN_2_O_	π_BNO_2__	π_BO_3__
BN	0.70	0.13	0.11	0.06
P-BN_IL-HF_	0.60	0.23	0.03	0.14
P-BN_IL-LF_	0.59	0.23	0.04	0.14
P-BN_PA-HF_	0.64	0.20	0.04	0.12
P-BN_PA-LF_	0.64	0.20	0.05	0.11

**Table 3:** –OB_3_ Species Concentration in Pristine BN and P-Doped BN Samples, as Derived from Double Integration of Simulated EPR Signal under Dark Conditions (Figures S9 and S10)^a^

sample	–OB_3_ concentration per g_sample_
BN	20
P-BN_IL-HF_	5550
P-BN_IL-LF_	650
P-BN_PA-HF_	1150
P-BN_PA-LF_	60

a Estimated error is 20%.

To further examine the chemical environment in the materials, we performed an XPS analysis. The B 1s, N 1s, O 1s, and C 1s XPS deconvolution spectra for pristine BN and P-BN samples can be found inFigure S3. By analyzing the B 1s and C 1s spectra in both pristine and P-doped BN, we observe similar chemical states for B (B–N and B–O–N bonds) and C. The deconvolution of the N 1s spectra indicates a preference for N–B and graphitic C–N bonds in P-BN, in contrast to N–B and B–O–N bonds in pristine BN.^63,64^ Furthermore, the second chemical state of O (aside from that of O–B) also exhibits differences between pristine and doped BN. To determine the chemical state of P, we conducted XPS measurements on the phosphazene reference sample and on the P-BN samples. The acquired P 2p XPS spectra are presented in Figure 3b. The P 2p core level of the phosphazene exhibits a doublet with the main peak positioned at 133.5 eV, which is characteristic of P–N bond.^23,24,27,65,66^ Again, the P-BN samples exhibit a similar chemical state for P, regardless of the P dopant source. The deconvoluted peaks, although similar, are shifted by approximately 0.1–0.7 eV compared with the phosphazene reference sample. This observation points to the creation of P–N or P=N bonds in the materials. The chemical environment surrounding the P atoms, as derived from XPS analysis, aligns with the previously discussed NEXAFS results. This helps us suggest a plausible chemical structure for the P-doped BN samples, illustrated in Figure 3c. Here, we propose that P atoms either “replace” B atoms and bond with three or four surrounding N atoms of the main BN structure and/or graft to the BN structure forming =NH and/or –NH_2_ groups outside the plane. The former configuration aligns with some theoretical studies that found this to be the most stable configuration. Our analyses do not support the replacement of N atoms by P atoms, as suggested by some former experimental work.

### 3.2 CO_2_ Adsorption

**Figure 4:**
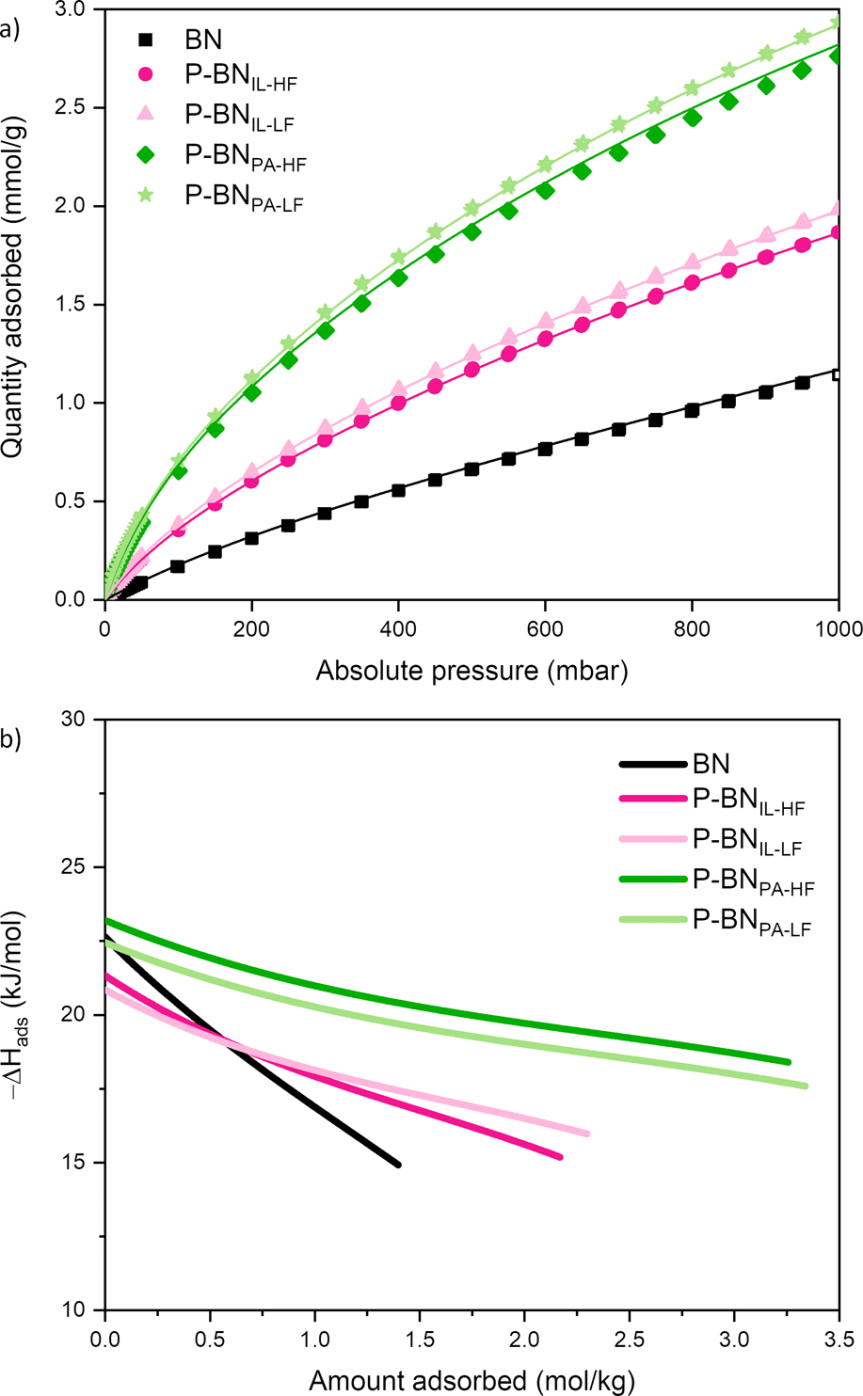
(a) Experimental CO_2_ adsorption (25 °C) isotherms for pristine BN and P-doped BN samples fitted using a dual site Langmuir fit. Symbols are used for the data points and solid line represents the isotherm fit; (b) isosteric heat of adsorption for pristine BN and P-doped BN samples plotted vs amount of loading. Heat of adsorption was calculated by fitting experimental CO_2_ adsorption data obtained at three temperatures (15, 25, and 35 °C) for all materials to the virial isotherm model.

To examine CO_2_ adsorption, we measured the CO_2_ uptake at 25 °C and up to 1 bar absolute pressure for pristine BN and P-doped BN samples. The obtained isotherms are shown in Figure 4a. We notice the following CO_2_ uptake trend at 1 bar: BN (1.13 mmol g^–1^) < P-BN_IL-HF_ (1.86 mmol g^–1^) < P-BN_IL-LF_ (1.98 mmol g^–1^) < P-BN_PA-HF_ (2.77 mmol g^–1^) < P-BN_PA-LF_ (2.93 mmol g^–1^). We conclude that P-doping, especially using phosphoric acid as the P precursor, noticeably enhances CO_2_ adsorption compared with pristine BN. Given the shape of the isotherm we do not link this to a chemisorption effect related to the presence of P. In fact, the CO_2_ uptake does not correlate the amount of O-containing or P-containing functional groups in the samples. Instead, the increasing trend in CO_2_ uptake seems to follow the increasing trend in the materials’ ultramicropore (≤0.7 nm) volume (Table S1). Studies have shown that ultramicropores favor CO_2_ adsorption.^67−69^ We conclude that structure/porosity plays a role in the CO_2_ sorption, with the ultramicropores having the main contribution. To provide perspective on the CO_2_ uptakes measured, we note that other studies on porous BN and BNO have reported CO_2_ uptake up to ∼1.6 mmol g^–1^, while reported BCN samples have shown an enhanced CO_2_ adsorption up to ∼3.8 mmol g^–1^ under the same conditions (25 °C, 1 bar).^3,9,57,58,70−72^

To understand more about adsorbate–adsorbent interactions, we measured CO_2_ adsorption at three different temperatures (15, 25, and 35 °C), and fitted the isotherms using the dual-site Langmuir model. The isotherms fits and model coefficients can be found in Figure S4 and Table S2, respectively. From the good fit of the isotherms, we conclude that this model describes the adsorption of CO_2_ on our materials well. Moreover, we calculated the isosteric heat of adsorption for each material by fitting our experimental CO_2_ adsorption equilibrium data (at 15, 25, and 35 °C) to the virial isotherm model, for which the coefficients are listed in Table S3. We plotted the calculated heat of adsorption values as a function of the CO_2_ loading for pristine BN and P-BN samples and present the results in Figure 4b. The results confirm that CO_2_ is physisorbed onto our materials and the heat of adsorption, especially in the case of doped BN, stays relatively constant as loading increases. At zero loading, the heats of adsorption for pristine and P-doped BN are similar (ranging between 20 and 25 kJ mol^−1^). Our heat of adsorption values broadly agree with reported values in literature on pristine porous BN (19–28 kJ mol^−1^), which are lower than in the case of BCN (32–35 kJ mol^−1^).^9,73,74^

### 3.3 Optoelectronic Properties

**Figure 5:**
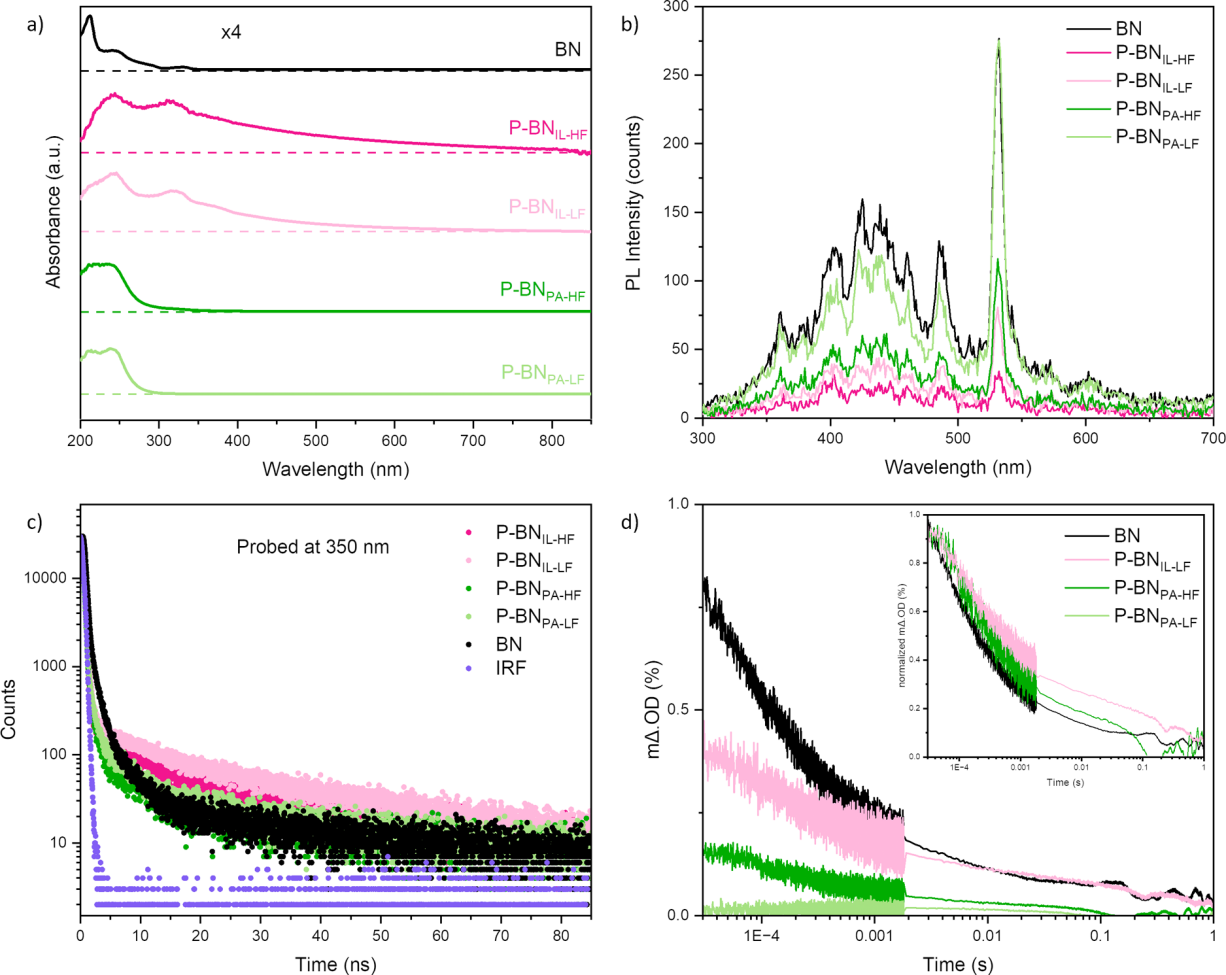
(a) UV–vis spectra, (b) steady-state PL spectra obtained upon excitation at 200 nm, (c) TCSPC decay profiles probed at 350 nm upon excitation at 282 nm, and (d) TAS decay profiles probed at 900 nm upon excitation at 355 nm of pristine BN and P-doped BN samples. Multiplication factors are shown on top of the absorption data in (a) to facilitate comparison between samples.

Understanding the light absorption behavior of a material is critical to understanding its ability to harvest sunlight. To study our materials’ light absorption behavior, we used UV–vis spectroscopy, with the data presented in Figure 5a. P-doping overall results in extended absorbance into the visible spectrum, with the choice of the P source showing differences. Using ionic liquid as the P precursor results in absorbance over the whole UV–vis spectrum (200–850 nm), potentially because of the higher C content.^75−77^ We observe tail states near the optical band edges of pristine BN and P-doped BN, which can be assigned as Urbach-tails, given their exponential character and the amorphous, defective nature of our materials.^78^

Steady-state PL spectra obtained upon excitation at 200 nm are shown in Figure 5b, detailing the radiative relaxation spectra for all of our materials. The radiative relaxation spectra are similar in pristine BN and P-doped BN. This indicates that electrons populate similar excited states upon irradiation, regardless of P-doping. Pristine BN shows the highest signal intensity followed by P-BN_PA-LF_, P-BN_PA-HF_, P-BN_IL-LF_, and P-BN_IL-HF_. This trend could be explained by either one or a combination of the following scenarios: (i) pristine BN has a higher charge recombination rate than P-BN, and/or (ii) photoexcited electrons in P-BN show a stronger tendency to relax nonradiatively, in a way that is not measured using PL. The lower intensity signal in P-BN samples indicates that band-to-band PL emission is lost as charges are trapped in midgap states, increasingly formed/populated with P-doping.

To elucidate the time-resolved behavior of these photoexcited species, we measured for the first time the PL decay kinetics by using TCSPC. The kinetics were measured using 282 nm LED excitation source and probed at 350 nm emission wavelength as shown in Figure 5c. We selected the probe wavelength based on steady-state PL spectra obtained after excitation at 282 nm and reported in Figure S5. The fitted decay profiles can be found in Figure S6. For all samples, the PL decay kinetics exhibit two decay components, as we previously reported for BN.^3^ In the case of P-doped BN, a fast exponential decay component in ps-ns time scale, which is faster than the time resolution of the instrument response (IRF ∼ 1.5 ns) limiting its analysis. The faster decay of P-doped BN relative to BN suggests: (i) faster exciton recombination or (ii) increased trapping of photoexcited charge carriers in P-doped BN materials, and therefore, an increased loss of the radiative decay pathway. At longer decay times (>10 ns), a second decay component dominates in the kinetics of all samples, which might indicate shallowly trapped charges in P-doped BN, consistent with those kinetics reported for BN and g-C_3_N_4_ materials.^3,79^ These results suggest that P-doping increases the number or population of midgap states in the material—and subsequently the number of trapped charges—and hence the longer lifetimes compared to pristine BN. Additionally, different P-precursors have different impact on optoelectronic properties, with the IL route resulting in more long-lived decays.

To provide further insight on the charge carrier behavior of our samples, we carried out for the first time TAS measurements using a 355 nm laser source, with transient absorption decay profiles probed at 900 nm over time presented in Figure 5d. The decay profiles differ between pristine and doped materials and also between P-BN_PA_ and P-BN_IL_. Within the time resolution of the measurement (10 μs), pristine BN shows larger transient absorption signals (mΔO.D) than all other samples, which indicates that this sample possesses a larger initial population of charge carriers: BN (∼0.8 mΔO.D) > P-BN_IL-LF_ (∼0.4 mΔO.D) > P-BN_PA-HF_ (∼0.2 mΔO.D) > P-BN_PA-LF_. However, the rate at which these charge carriers recombine differs, with P-BN samples showing longer half-times (i.e., the time until the initial transient absorption signal seen at 10 μs reaches 50% of its initial value): BN (90 μs) < P-BN_PA-HF_ (480 μs) < P-BN_IL-LF_ (570 μs) < P-BN_PA-LF_. The half-times of our materials are comparable to those of pristine TiO_2_ and TiO_2_-MOF heterojunctions (∼180 μs),^3,79−81^ and longer than some TiO_2_ nanosheets, Bi_2_WO_6_–TiO_2_ heterojunctions (1–1.5 ns),^82^ and g-C_3_N_4_ and g-C_3_N_4_-based materials (10–100 μs).^75,79,83^ It should be noted that the TAS spectra notably differ between pristine BN and P-BN samples (Figure S7), where bleaching processes are primarily only seen in the blue-green region of the electromagnetic spectrum in pristine BN but further into the red in P-BN. The observation of a bleach is due to the loss in ground state absorption, and the fact that this extends further into the red for P-BN is due to the red-shift in band gap due to P-doping, with similar behavior seen in N-doped TiO_2_ previously.^84^ Interestingly, many of these bleach states transition to a positive signal at later times (typically from ∼0.1 to 1 ms), which indicates that the potential energies of the charge carriers formed in these materials change with time. As the bleach in the blue region is consistently the slowest to recover (Figure S8), this indicates that the potential energy of charge carriers reduces with time as they become increasingly more deeply trapped (as the energy required to probe them increases).

Overall, our TAS results support our TCSPC measurements in that they both show that BN possesses the highest populations of charge carriers (at the resolution of each measurement) yet P-doping causes the rate of recombination in these materials to slow. This, we propose, is likely due to the formation of midgap states in P-BN, which trap charge carriers more deeply, slowing their recombination yet likely making them less mobile.

**Figure 6:**
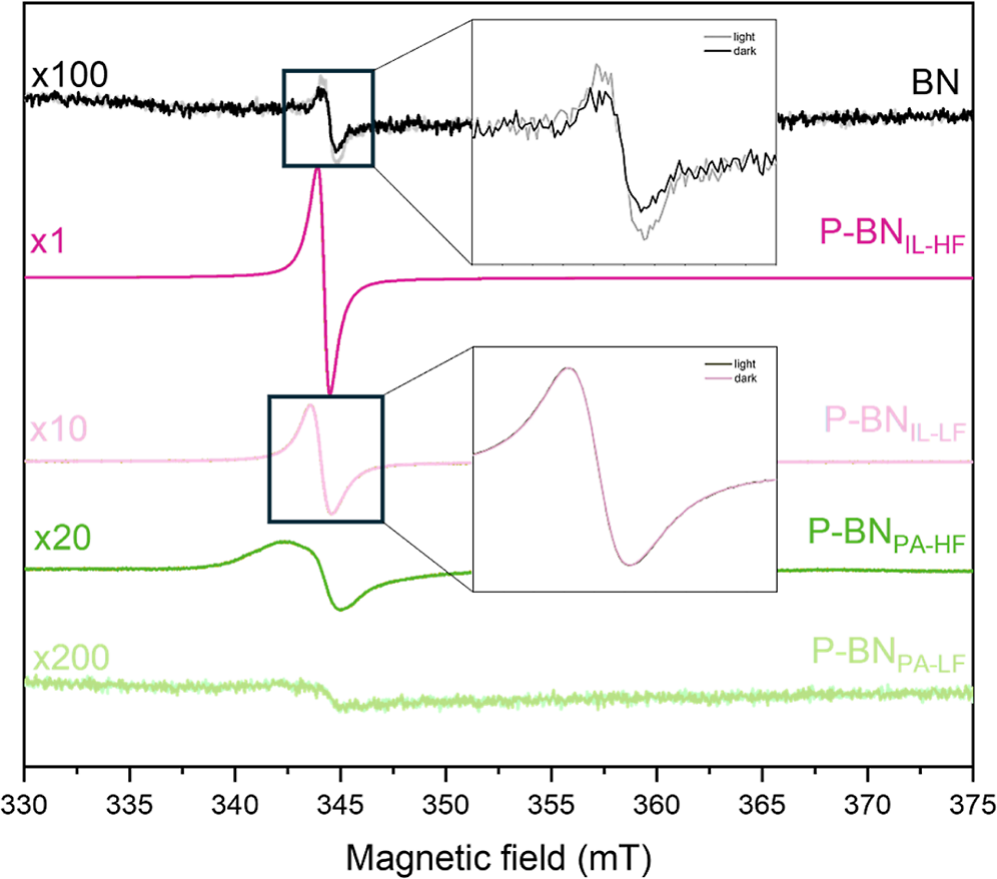
EPR spectra of pristine BN and P-doped BN samples in ambient temperature and an air atmosphere. Multiplication factors are shown on the left side of the signals to facilitate comparison between samples. Zoomed in graphs of pristine BN (up) and P-BN_IL-LF_ (below) spectra indicate change in signal before (darker color) and after (lighter color) irradiation with UV light source.

We used EPR spectroscopy to further probe the electronic properties of our materials. The EPR signal intensity is proportional to the concentration of unpaired electrons. We show the signal change before and after irradiation with a UV light source over continuous scanning of the magnetic field in Figure 6. Previous studies on porous BN suggest that unpaired electrons located in the paramagnetic –OB_3_ bonding pattern contribute to the EPR signal.^3,19,85^ P-BN_PA_ samples show a broader signal compared to that of pristine BN and P-BN_IL_ samples. This feature suggests that, in P-BN_PA_, the paramagnetic –OB_3_ radical is less delocalized. This could lead to a partially resolved hyperfine interaction with nearby nuclei or a possible tilt with respect to the molecular frame. We simulated the EPR spectra of our samples under dark conditions, and the experimental/simulated EPR signal comparison is shown in Figure S9. For pristine BN and P-BN_IL_ samples, the simulations provided a good fit to the experimental data, while for P-BN_PA-HF_ sample, the fitting error was larger (due to the broader signal). By integrating the simulated EPR signal of our samples, we obtained the EPR absorption response for each sample, presented in Figure S10. We further integrated the absorption signal and calculated the concentration of –OB_3_ species in our samples, which we assume are the main contribution to the EPR signal. We present the calculated concentrations, with approximately 20% error, in Table 3 below. The –OB_3_ concentration follows the trend: P-BN_IL-HF_ > P-BN_PA-HF_ > P-BN_IL-LF_ > P-BN_PA-LF_ > BN, suggesting that all P-BN samples have more defect sites than pristine BN, with the N_2_ flow rate during synthesis being the most influencing factor on defects creation. Upon UV irradiation, pristine BN shows an increase in the signal intensity, which could be attributed to photoexcited electron–hole pairs.^3^ However, we do not spot a difference in the signal in any of the P-BN samples before and after illumination, meaning there are no photoexcited electrons detected (or their concentration is too low to be detected).

**Figure 7:**
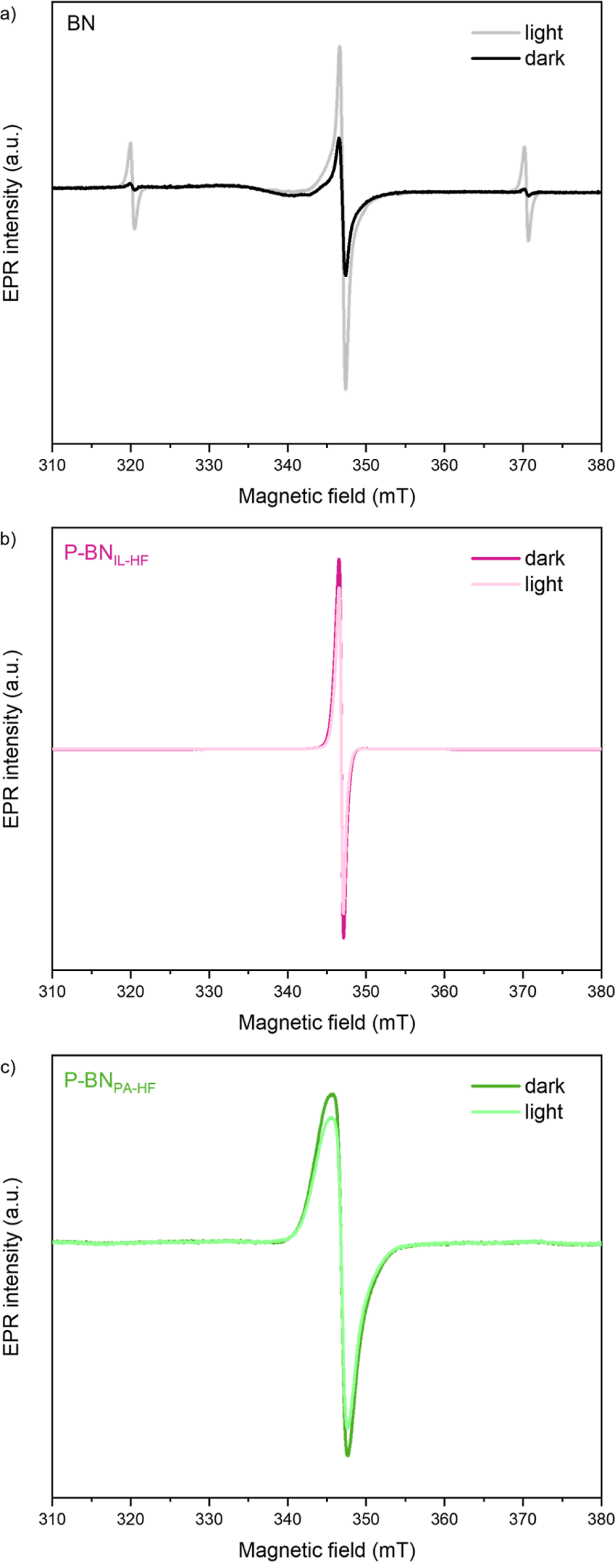
EPR spectra of (a) pristine BN, (b) P-BN_IL-HF_, and (c) P-BN_PA-HF_ at −268 °C and He atmosphere, indicating change in signal before (darker color) and after (lighter color) irradiation with UV light source.

To provide a second level of confidence with regard to the existence of photoexcited electrons in our samples, we conducted EPR measurements at −268 °C, with and without irradiation, on three selected samples: BN, P-BN_IL-HF_, and P-BN_PA-HF_. The results obtained through these measurements are presented individually in Figure 7, and comparatively to each other in Figure S11. As shown in Figure 7a, under dark conditions and at −268 °C, pristine BN exhibits a strong central peak along with two extra smaller peaks (splitting around 700 MHz) potentially caused by a hyperfine interaction with a ^14^N atom. Under irradiation, the intensity of all three peaks increases significantly, confirming the existence of photoexcited electrons. Even after switching the irradiation off, the increased signal persists, supporting these light-induced excited electrons are stable and long-lived. On the other hand, the EPR signal of P-BN samples (Figure 7b,c) before illumination shows very little difference between room temperature and −268 °C spectra. Interestingly, upon illumination, the EPR signal of both P-BN samples decreases. By performing thermal reversibility tests—heating up the sample to room temperature and then cooling it back down to −268 °C—we observe that (i) the EPR signal increases back to higher than the original intensity under dark, and (ii) the EPR signal decrease is photoinduced and not permanent. Therefore, we exclude the scenario of our P-BN materials degrading under light, and we assign the EPR signal decrease to partial photoinduced electron transfer from paramagnetic –OB_3_ species to surrounding atoms in the structure. This effect on EPR signal upon irradiation has been reported in literature, in molybdenum–copper (Mo–Cu) complexes, and attributed to (i) spin transition on the Mo center and (ii) electron transfer from Mo to Cu atoms, changing their oxidation states.^86^ From our EPR data, we can conclude that P-BN samples: (i) indeed have higher concentration of defects compared to pristine BN, as suggested by our XPS and NEXAFS results, and (ii) do not clearly exhibit creation of photoexcited electrons upon irradiation, as opposed to pristine BN.

### 3.4 CO_2_ Photoreduction

**Figure 8:**
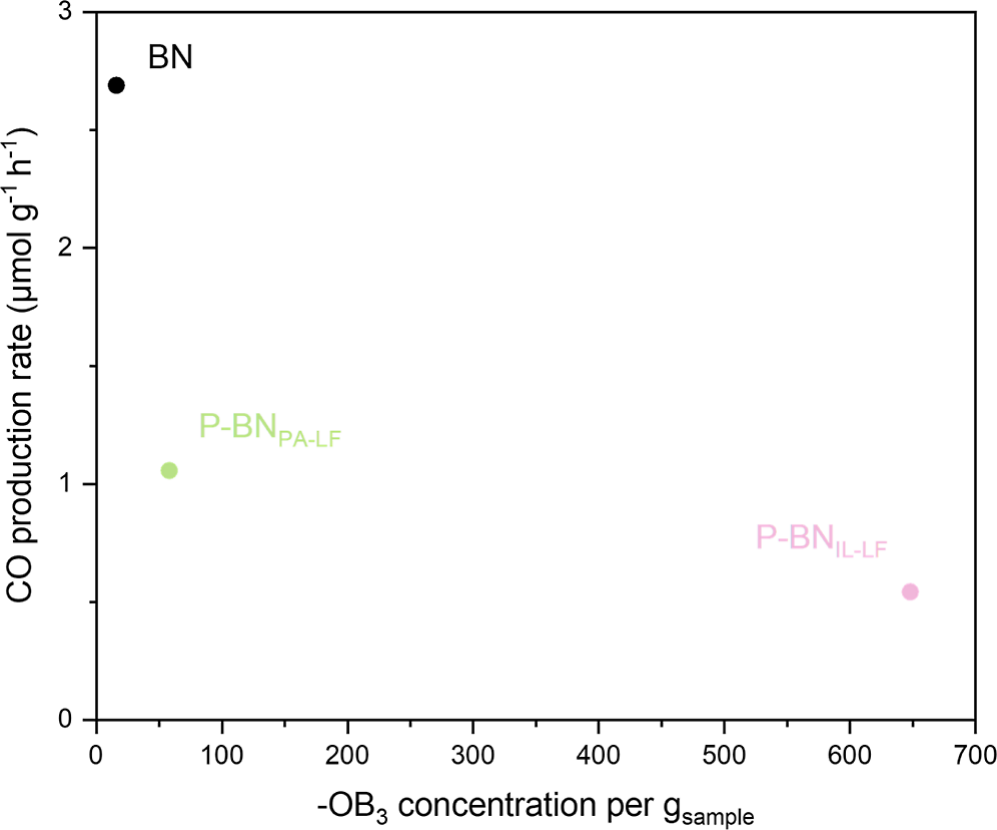
Effect of the –OB_3_ species concentration in pristine BN and P-doped BN samples (as derived from simulated EPR spectra before irradiation) on the photocatalytic production of CO (using CO_2_/H_2_ as feed, under ∼1 sun irradiation, 5 h reaction).

We tested our pristine BN and P-BN materials for photocatalytic CO_2_ reduction using H_2_ as a sacrificial agent in a gas–solid reaction. The products of this reaction are CO (main product) and CH_4_ in different ratios for pristine BN and P-BN. Full details on CO and CH_4_ production rates under all testing conditions including control tests can be found in Table S4 and Figure S12. Interestingly, previous studies on CO_2_ photoreduction using pristine and Cu-doped BN^3,87^ as well as Pd_*x*_Cu_*y*_-BN alloys^88^ pointed to the dominant photocatalytic activity of the materials. Yet, here, control tests point to the fact that a majority of the CO and CH_4_ production of all BN samples (especially P-doped) comes from degradation of the material upon irradiation or from side-reactions with H_2_, and only a small portion comes from photocatalytic reduction of CO_2_ gas. Partial degradation of the porous BN has been reported before^3^ and further work would be needed to understand its mechanism and how P doping might influence it. This poor photocatalytic activity may be explained by our TCSPC, TAS, and EPR results (Section 3.3), which indicated P-doping results in an increased concentration of chemical defects (such as higher O content) and, as a result, midgap states that more deeply trap charge carriers and therefore hinder (i) their ability to transport to catalytic sites and (ii) the potential energy to drive CO_2_ reduction. As a fact, through our EPR measurements, we show that the concentration of –OB_3_ species increases from BN < P-BN_PA-LF_ < P-BN_IL-LF_, which follows the reverse trend to CO_2_ photoreducing activity, where BN > P-BN_PA-LF_ > P-BN_IL-LF_. The relationship between the –OB_3_ concentration and photocatalytic activity is shown in Figure 8. We note that, based on literature, the presence of O (up to 10 at. %) in the pristine porous BN structure has a positive effect on the material’s CO_2_ photoreducing activity.^3^ Therefore, the drop in photocatalytic activity from BN to P-BN samples should be attributed to the integration of P in the structure and to the resulting increased number of defects (including, but not limited to, higher O content) and more/more populated midgap states. The connection between midgap states (acting as trapping sites) caused by structural defects and photoactivity has been previously reported for other materials in the literature, and pinpoints the importance of thermodynamics and the energetic distribution of midgap states facilitating photoactivity.^89−91^

## 4 Conclusions

Herein, we successfully doped P into the structure of porous amorphous BN by using two different P-containing precursors: phosphoric acid and an ionic liquid. We report for the first time a detailed, combined study on how P-doping modified the chemistry and porosity of the material, leading to changes in sorptive, optoelectronic, and photocatalytic properties. The changes are apparent not only between pristine and doped samples but also between the doped samples from different P-precursors.

From a materials chemistry point of view, in-depth XPS and NEXAFS analyses suggest that P forms bonds with surrounding N atoms, along with –NH_2_ and/or =NH groups between planes in a tetrahedral configuration. This is partly in accordance with theoretical studies in the literature, which suggest that the P incorporation in the BN structure would cause distortion and that the P_B_ replacement would be the most thermodynamically favorable placement. While the amorphous BN structure inherently comes with defects (such as N vacancies, B and N substitutions with C and/or O, and creation of –OH groups on the edges), P-doping causes further structural defects (such as P incorporation) and increases C and O content, particularly when using ionic liquid as precursor. Resulting from these structural changes, P-doped BN samples have more ultramicropores than do pristine BN, leading to enhanced CO_2_ physisorption, especially in samples prepared using phosphoric acid.

Together, the NEXAFS and EPR results indicate that the defects in P-doped BN samples increase the concentration of unpaired electrons. Based on EPR results, pristine BN seems to create more unpaired electrons upon irradiation than P-doped BN. In addition to this, PL, TCSPC, and TAS analyses show that the creation of more defects in the structure by P-doping (especially when using ionic liquid precursor) results in midgap states which act as electron traps. While this leads to longer lifetimes for the charged species (slower recombination rates), their reaction with CO_2_ and H_2_ for photocatalytic CO_2_ reduction will likely be hindered because of the loss in mobility and potential energy of charge carriers that populate these states. Nonetheless P-doping extends light harvesting into the visible as well as the lifetime of charge carriers, which is necessary to drive kinetically challenging catalytic conversions, such as CO_2_ reduction. Therefore, we propose that future studies of this materials system focus on how the activity of these longer-lived states may be improved, through the use of codoping strategies and/or surface cocatalysts.

## Supporting Information Available

Details of reference samples, NEXAFS results, XPS spectra, PL spectra, TCSPC results, TAS spectra, EPR simulations and spectra, CO_2_ adsorption, and photocatalytic data (PDF)

## Abbreviations

BN: boron nitride
P-BN: phosphorus-doped boron nitride
PA: phosphoric acid
IL: ionic liquid
FTIR: Fourier transform infrared spectroscopy
ATR: attenuated total reflectance
XPS: X-ray photoelectron spectroscopy
NEXAFS: near edge X-ray absorption fine structure spectroscopy
TEY: total electron yield
XRD: X-ray diffraction
BET: Brunauer–Emmett–Teller
DRS-UV/vis: ultraviolet–visible diffuse reflectance spectroscopy
PL: photoluminescence
PMT: photomultiplier tube
TCSPC: time-correlated single photon counting
IRF: instrument response factor
TAS: transient absorption spectroscopy
EPR: electron paramagnetic resonance
DSL: dual-site Langmuir
GCMS: gas chromatographer–mass spectrometer
