# Supplementary material for: Effects of Phosphorus Doping on Amorphous Boron Nitride’s Chemical, Sorptive, Optoelectronic, and Photocatalytic Properties
Source: J Phys Chem C Nanomater Interfaces. 2024 Jul 24;128(31):13249–63. doi: 10.1021/acs.jpcc.4c02314 (PMC11317980; doi:10.1021/acs.jpcc.4c02314)
Supplement: Supplementary file 1 — jp4c02314_si_001.pdf [file jp4c02314_si_001.pdf]

Supporting Information

Effects of Phosphorus Doping on Amorphous Boron Nitride's Chemical, Sorptive, Optoelectronic and Photocatalytic Properties

Ioanna Itskou[1], Andreas Kafizas[2,3], Irena Nevjestic[3,4], Soranyel Gonzalez Carrero[5], David C. Grinter[6], Hassan Azzan[1], Gwilherm Kerherve[4], Santosh Kumar[6], Tian Tian[1†], Pilar Ferrer[6], Georg Held[6], Sandrine Heutz[3,4], Camille Petit*[1]

[1] Barrer Centre, Department of Chemical Engineering, Imperial College London, London SW7 2AZ, U.K.

[2] Department of Chemistry, Molecular Sciences Research Hub, Imperial College London, London W12 7TA, U.K.

[3] London Centre for Nanotechnology, Imperial College London, London SW7 2AZ, U.K.

[4] Department of Materials, Imperial College London, London SW7 2AZ, U.K.

[5] Department of Chemistry, Centre for Processable Electronics, Imperial College London, London W12 7TA, U.K.

[6] Diamond Light Source, Harwell Science and Innovation Campus, Didcot OX11 0DE, U.K.

[†] Current address: Department of Applied Biology and Chemical Technology, The Hong Kong Polytechnic University, Hung Hom, Hong Kong

* Corresponding author

Table S1. Textural parameters of pristine BN and P-doped BN, as derived from N_2 sorption isotherms (- 196 °C). S_{BET} indicates BET surface area, V_{tot} is the total pore volume, V_{meso} is the volume of mesopores, V_{micro} is the volume of micropores, and V_{ultra} is the volume of ultramicropores (≤ 0.7 nm).

**Sample**	**S_{BET} (m^2 g^{-1})**	**V_{tot} (cm^3 g^{-1})**	**V_{meso} (cm^3 g^{-1})**	**V_{micro} (cm^3 g^{-1})**	**V_{micro}/V_{tot} (%)**	**V_{ultra} (cm^3 g^{-1})**
BN	1100	0.577	0.151	0.426	74	0.084
P-BN_{IL-HF}	1276	0.652	0.162	0.490	75	0.131
P-BN_{IL-LF}	1314	0.674	0.175	0.499	74	0.129
P-BN_{PA-HF}	991	0.479	0.082	0.397	83	0.179
P-BN_{PA-LF}	1036	0.508	0.099	0.409	81	0.184

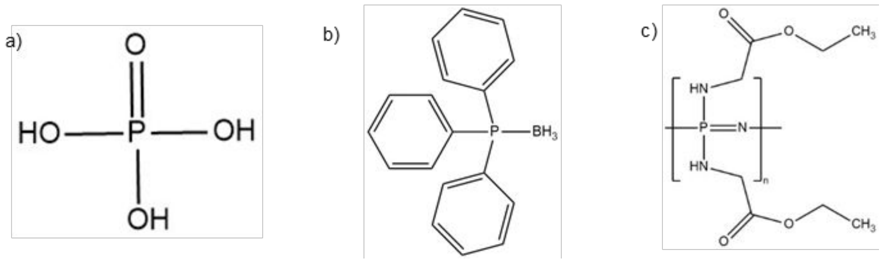

Figure S1. Reference samples for NEXAFS and XPS analyses. a) Phosphoric acid, b) borane triphenylphosphine complex ($(C_6H_5)_3P{\cdot}BH_3$), and c) poly(bis(1-(ethoxycarbonyl)methylamino)phosphazene ($(C_8H_{16}N_3O_4P)_n$).

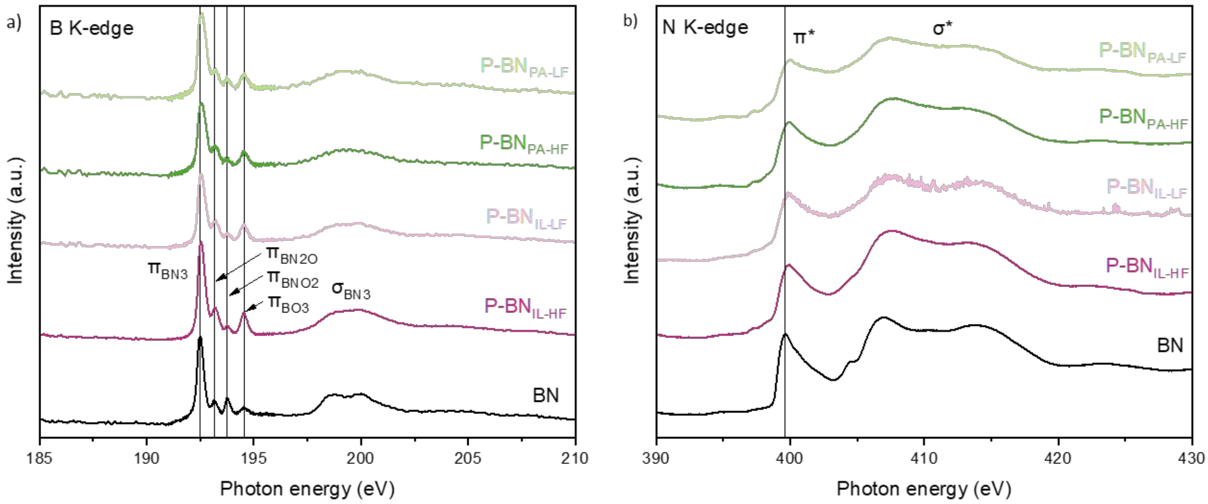

Figure S2. a) B K-edge and b) N K-edge NEXAFS spectra of pristine BN and P-doped BN samples with highlighted peaks[1,2]. It needs to be noted that this pristine BN was prepared using different synthesis precursors (boric acid + urea) than the pristine BN in main manuscript (boric acid + melamine). However, we have included it as a reference pristine sample since the two pristine BN samples share the same chemical bonds and environment.

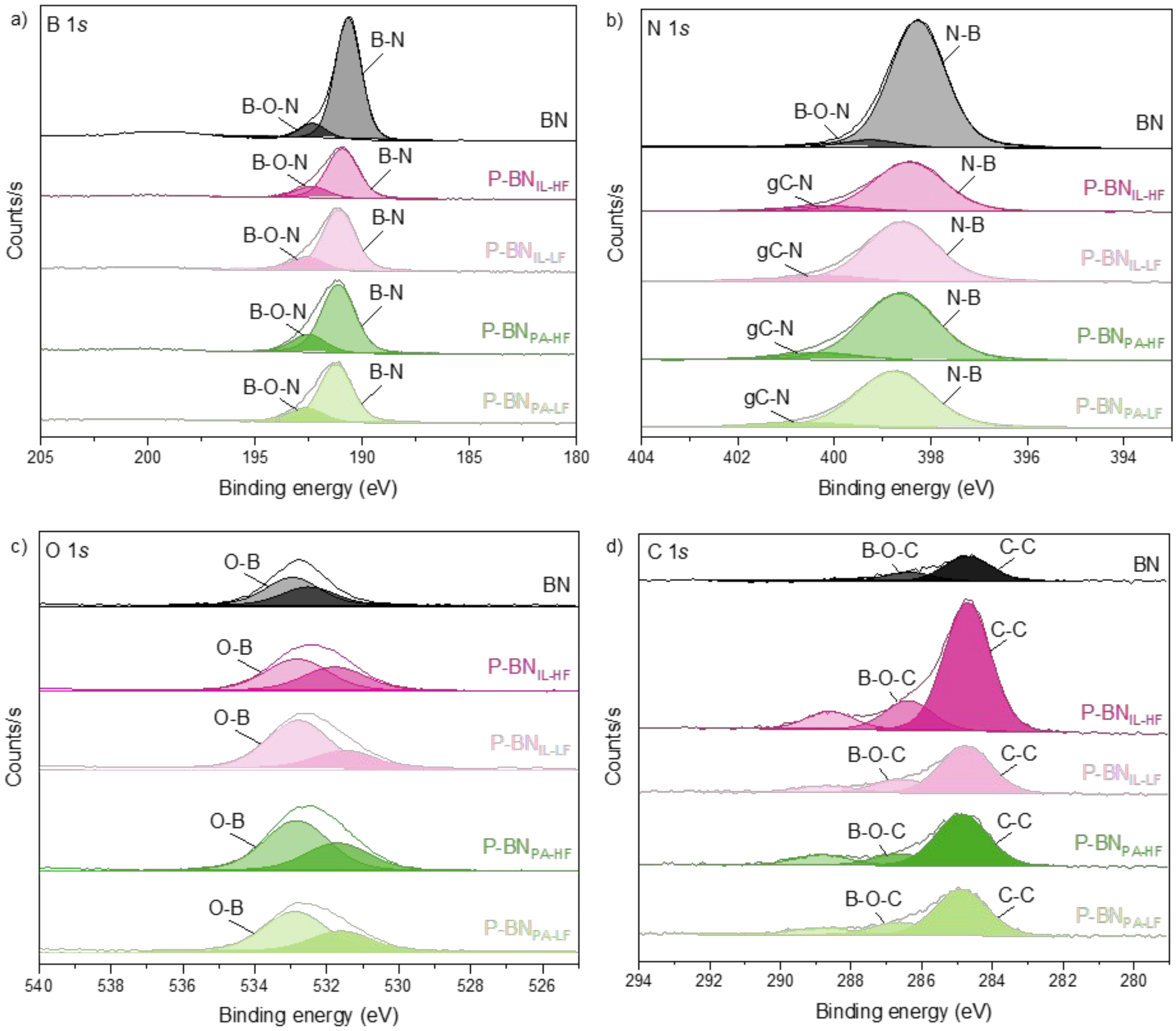

Figure S3. a) B 1*s*, b) N 1*s*, c) O 1*s* and d) C 1*s* XPS deconvolution spectra of pristine BN and P-doped BN samples. B 1*s* spectra indicate creation of B-N (~191 eV) and B-O-N (~192.5 eV) bonds in all samples. From N 1*s* spectra the peak appearing at ~398.5 eV in pristine and P-doped BN is assigned to N-B. The second chemical state of N in pristine BN is O-N bonding (~399 eV), while in P-BN it is gC-N (~400 eV). O 1*s* spectra of all samples show same O-B (~533 eV) bonding. In P-BN the second chemical state is shifted to ~531.5 eV. All samples show the same C chemical states from C 1*s* spectra, containing C-C (~284.8 eV) and O-C (~286.5 eV) bonds[1,3].

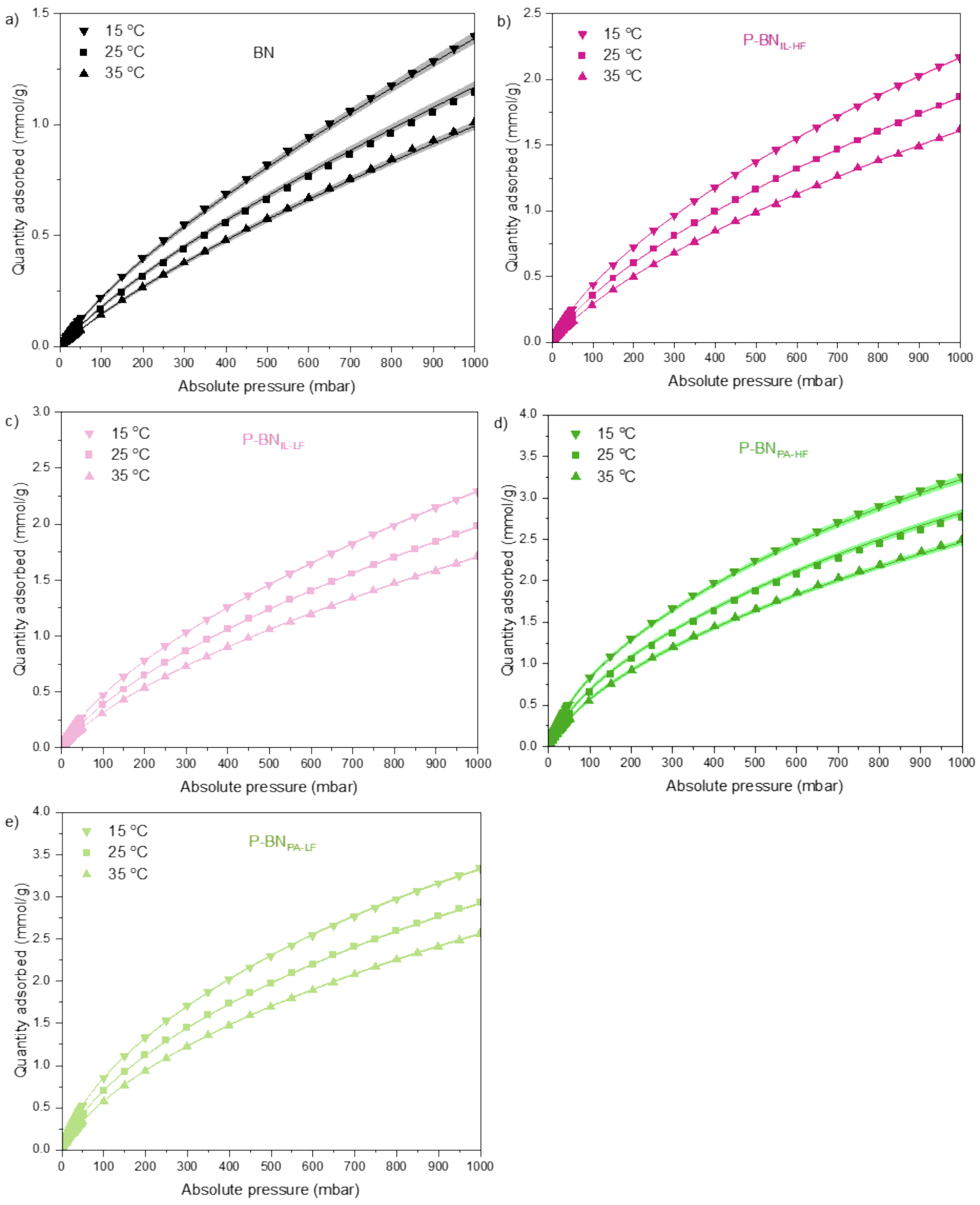

Figure S4. Experimental CO_2 adsorption isotherms taken at three temperatures (15, 25, 35 ^oC) and fitted using dual site Langmuir fit for a) pristine BN, b) P-BN_{IL-HF}, c) P-BN_{IL-LF}, d) P-BN_{PA-HF}, and e) P-BN_{PA-LF}. Symbols are used for the data points, solid line represents the isotherm fit and shading indicates confidence bounds.

Table S2. Dual site Langmuir (DSL) coefficients as derived from fitting adsorption data for pristine BN and P-doped BN samples. The values in parentheses represent the uncertainty values.

DSL coefficient	BN	P-BN$_{IL-HF}$	P-BN$_{IL-LF}$	P-BN$_{PA-HF}$	P-BN$_{PA-LF}$
q_{sb} (mol kg^{-1})	0.35 (0.01)	0.36 (0.00)	0.40 (0.00)	0.73 (0.01)	0.65 (0.00)
q_{sd} (mol kg^{-1})	17.98 (0.14)	6.51 (0.01)	6.83 (0.01)	6.55 (0.05)	6.41 (0.01)
b_0 (bar^{-1})	$9.19 \cdot 10^{-4}$ ($4.98 \cdot 10^{-5}$)	$2.97 \cdot 10^{-4}$ ($5.17 \cdot 10^{-6}$)	$3.06 \cdot 10^{-4}$ ($4.32 \cdot 10^{-6}$)	$14 \cdot 10^{-4}$ ($9.54 \cdot 10^{-5}$)	$4.17 \cdot 10^{-4}$ ($6.95 \cdot 10^{-6}$)
d_0 (bar^{-1})	$1.09 \cdot 10^{-4}$ ($9.08 \cdot 10^{-7}$)	$3.87 \cdot 10^{-4}$ ($6.60 \cdot 10^{-7}$)	$3.80 \cdot 10^{-4}$ ($5.52 \cdot 10^{-7}$)	$2.70 \cdot 10^{-4}$ ($2.73 \cdot 10^{-6}$)	$3.44 \cdot 10^{-4}$ ($7.57 \cdot 10^{-7}$)
$-\Delta U_b$ (kJ mol^{-1})	20.17 (0.14)	25.40 (0.04)	25.31 (0.04)	22.11 (0.16)	25.56 (0.04)
$-\Delta U_d$ (kJ mol^{-1})	15.32 (0.21)	16.58 (4.22)	16.63 (3.60)	18.60 (0.03)	18.36 (0.05)

Table S3. Virial coefficients as derived from calculations of isosteric heat of adsorption for pristine BN and P-doped BN samples. The values in parentheses represent the uncertainty values. The virial fit was used on experimental data from CO_2 adsorption isotherms for each material in three temperatures, which were shown in Fig. S4.

**Virial coefficient**	**BN**	**P-BN$_{IL-HF}$**	**P-BN$_{IL-LF}$**	**P-BN$_{PA-HF}$**	**P-BN$_{PA-LF}$**
a_0 (K)	-2722.5 (2.08)	-2565.8 (1.90)	-2507.7 (1.51)	-2790.7 (3.09)	-2700.2 (1.63)
a_1 (K mol^{-1})	850.07 (3.17)	562.29 (2.03)	459.37 (1.43)	351.09 (1.92)	343.51 (0.99)
a_2 (K mol^{-2})	-219.89 (2.90)	-195.06 (1.31)	-165.99 (0.85)	-98.51 (0.76)	-94.88 (0.39)
a_3 (K mol^{-3})	61.90 (2.44)	42.87 (0.76)	33.61 (0.46)	13.87 (0.27)	13.31 (0.14)
b_0 (-)	8.39 (0.01)	6.95 (0.01)	6.68 (0.01)	6.85 (0.01)	6.51 (0.01)
b_1 (mol^{-1})	-1.78 (0.01)	-0.62 (0.01)	-0.35 (0.01)	-0.10 (0.01)	-0.10 (0.00)

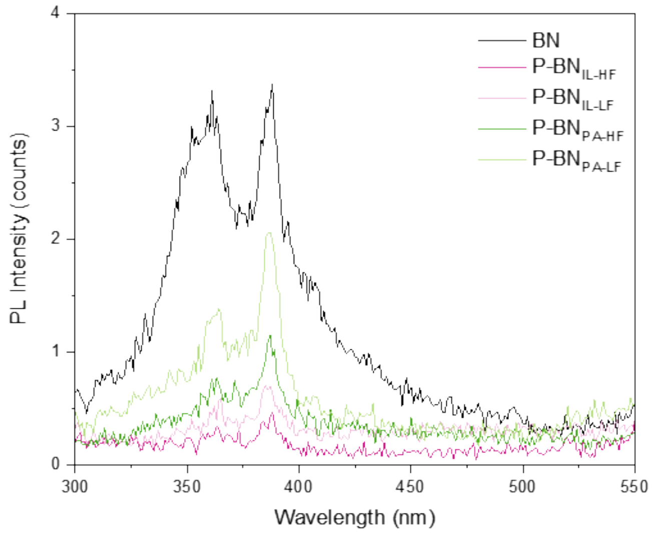

Figure S5. Steady-state PL spectra of pristine BN and P-doped BN samples obtained upon excitation at 282 nm.

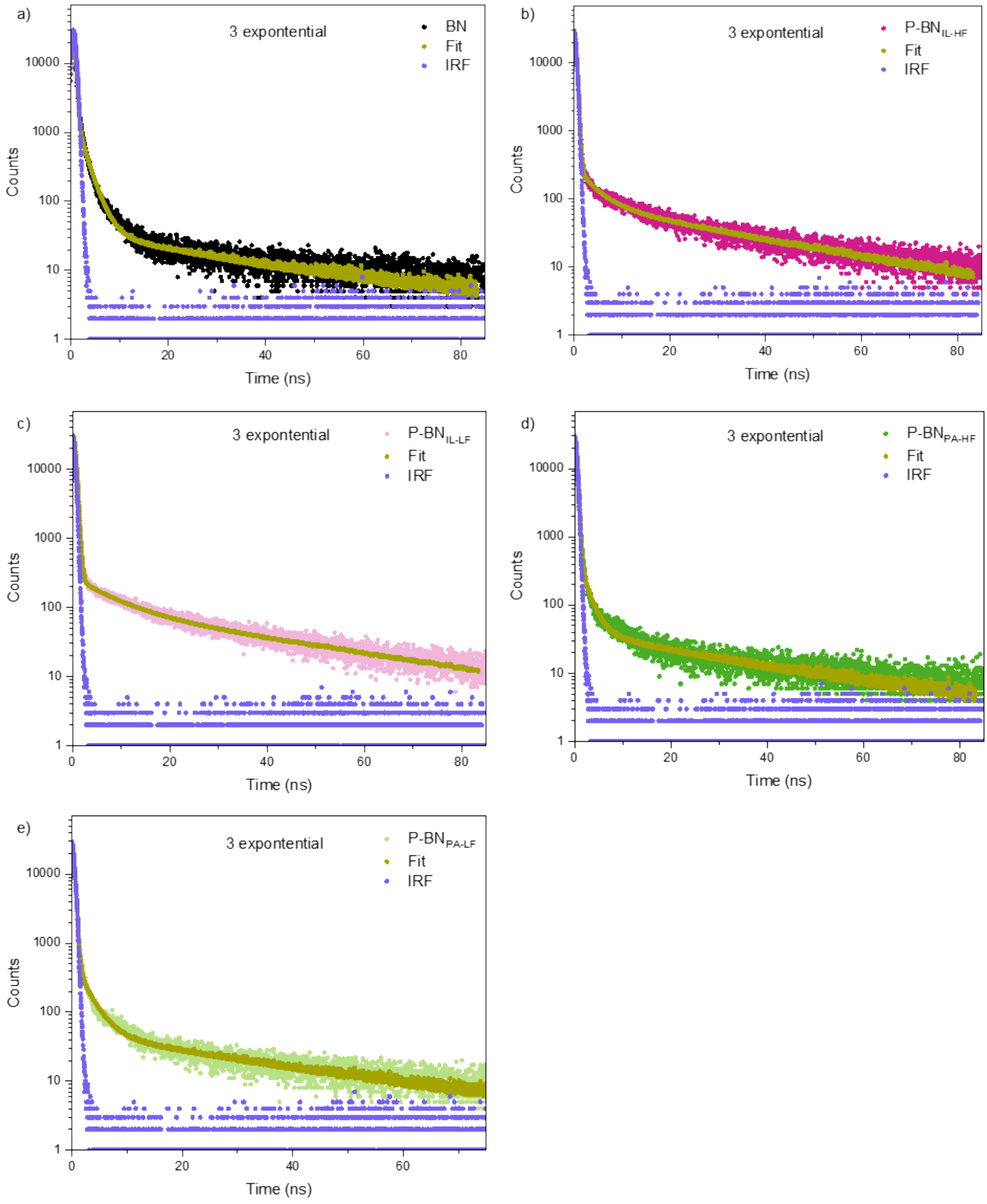

Figure S6. TCSPC decay profiles probed at 350 nm and fitted using triexponential function for a) pristine BN, b) P-BN$_{IL-HF}$, c) P-BN$_{IL-LF}$, d) P-BN$_{PA-HF}$, and e) P-BN$_{PA-LF}$. Shown in the plots along with the sample signal are the fit (thin line) and instrument response factor (IRF). Due to the fast decay being faster than our IRF we were unable to resolve the kinetics.

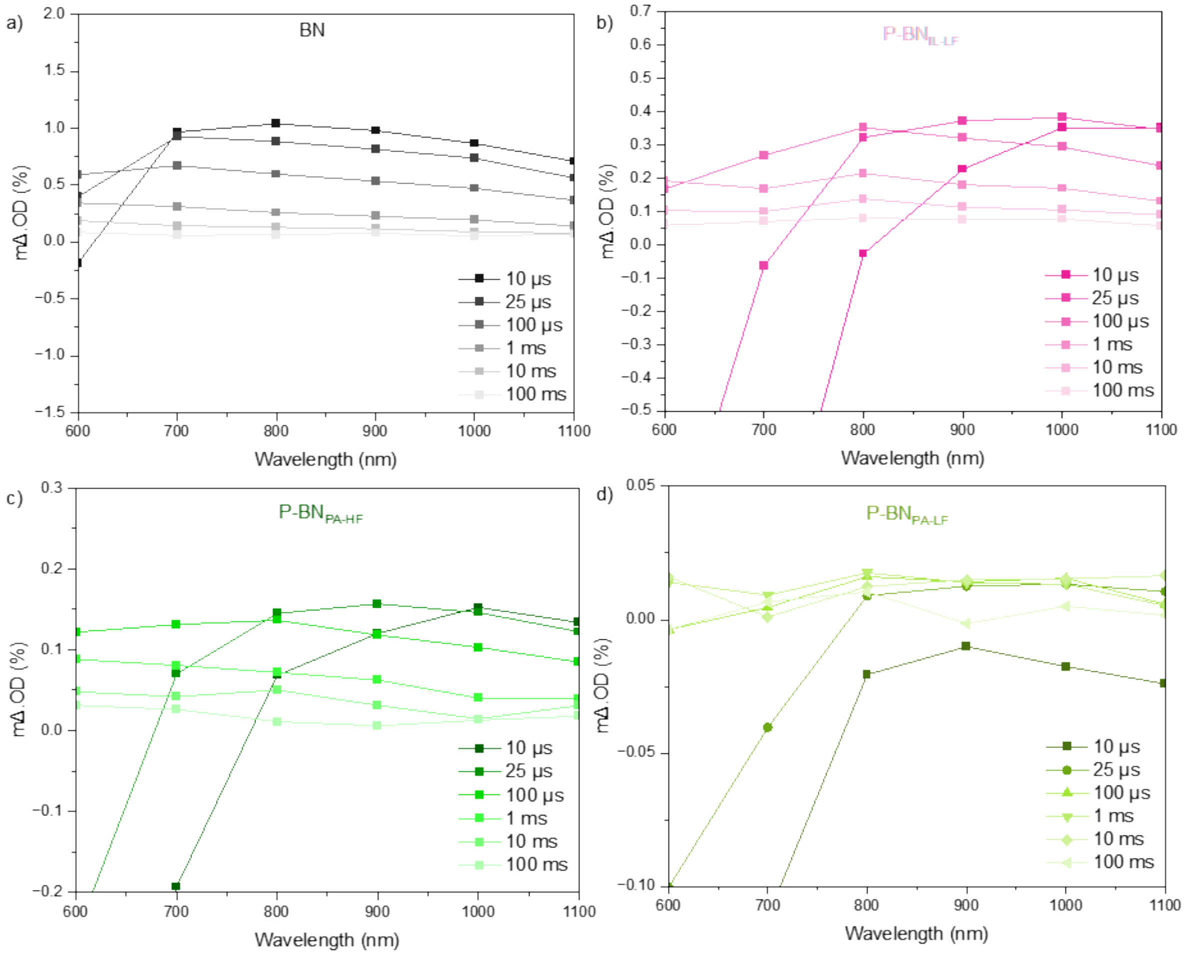

Figure S7. TAS data probed at a range 600 – 1100 nm of wavelengths upon excitation at 355 nm for a) pristine BN, b) P-BN$_{IL\text{-}LF}$, c) P-BN$_{PA\text{-}HF}$, and d) P-BN$_{PA\text{-}LF}$.

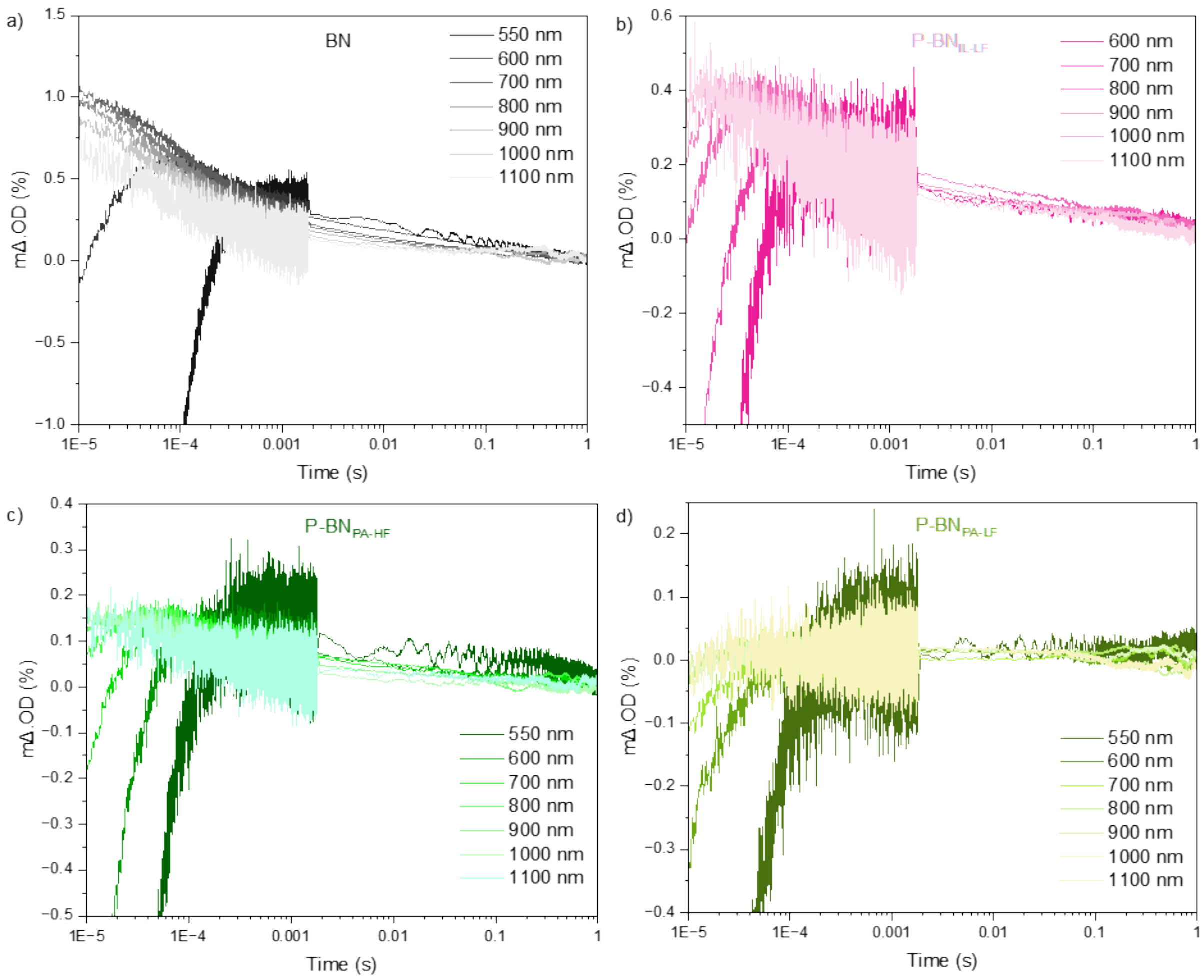

Figure S8. TAS decay profiles over time upon excitation at 355 nm for a) pristine BN, b) P-BN$_{IL-LF}$, c) P-BN$_{PA-HF}$, and d) P-BN$_{PA-LF}$.

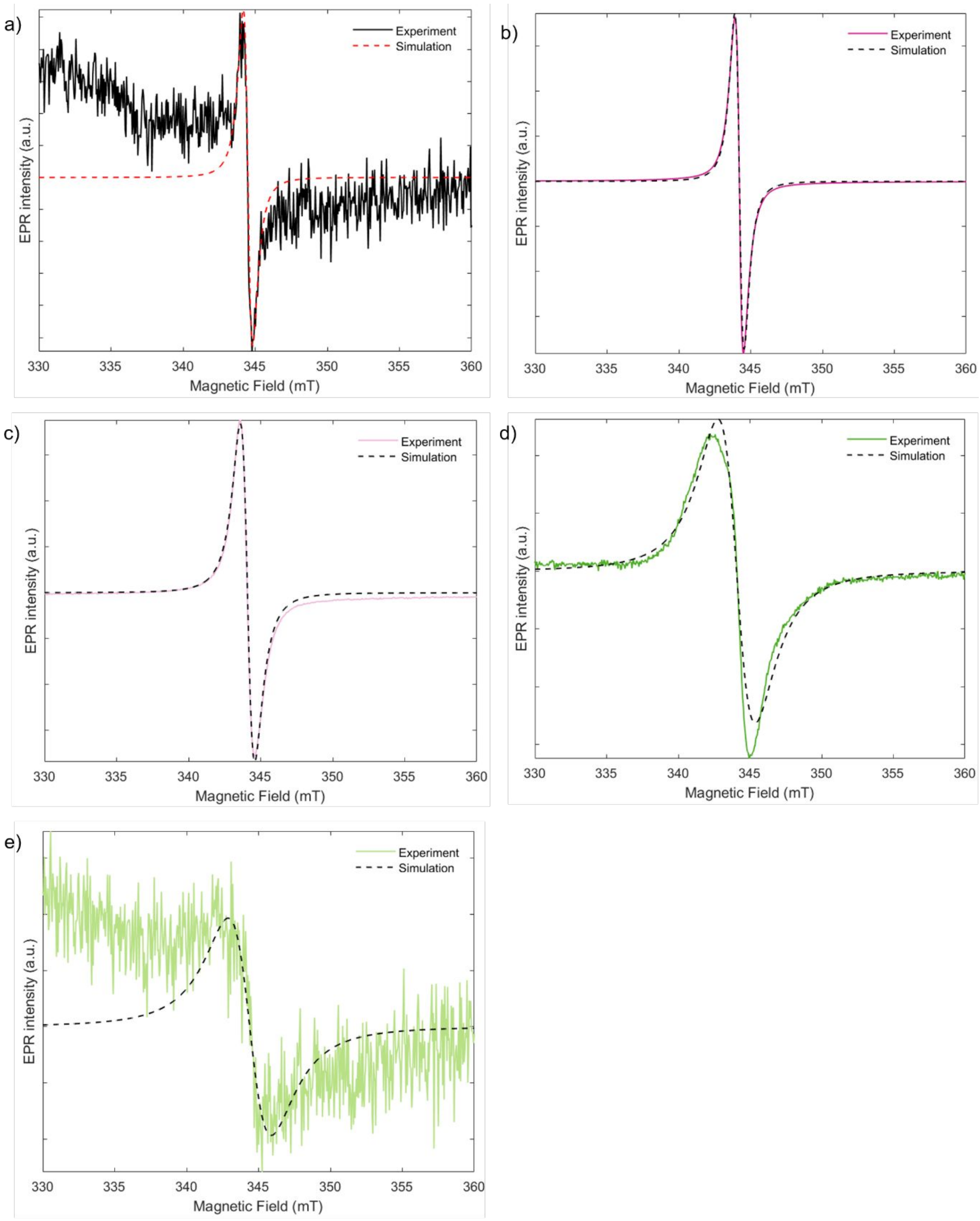

Figure S9. Experimental (solid line) and simulated (dashed line) EPR spectra obtained at room temperature and air atmosphere, of a) pristine BN, b) P-BN$_{IL\text{-}HF}$, c) P-BN$_{IL\text{-}LF}$, d) P-BN$_{PA\text{-}HF}$, and e) P-BN$_{PA\text{-}LF}$.

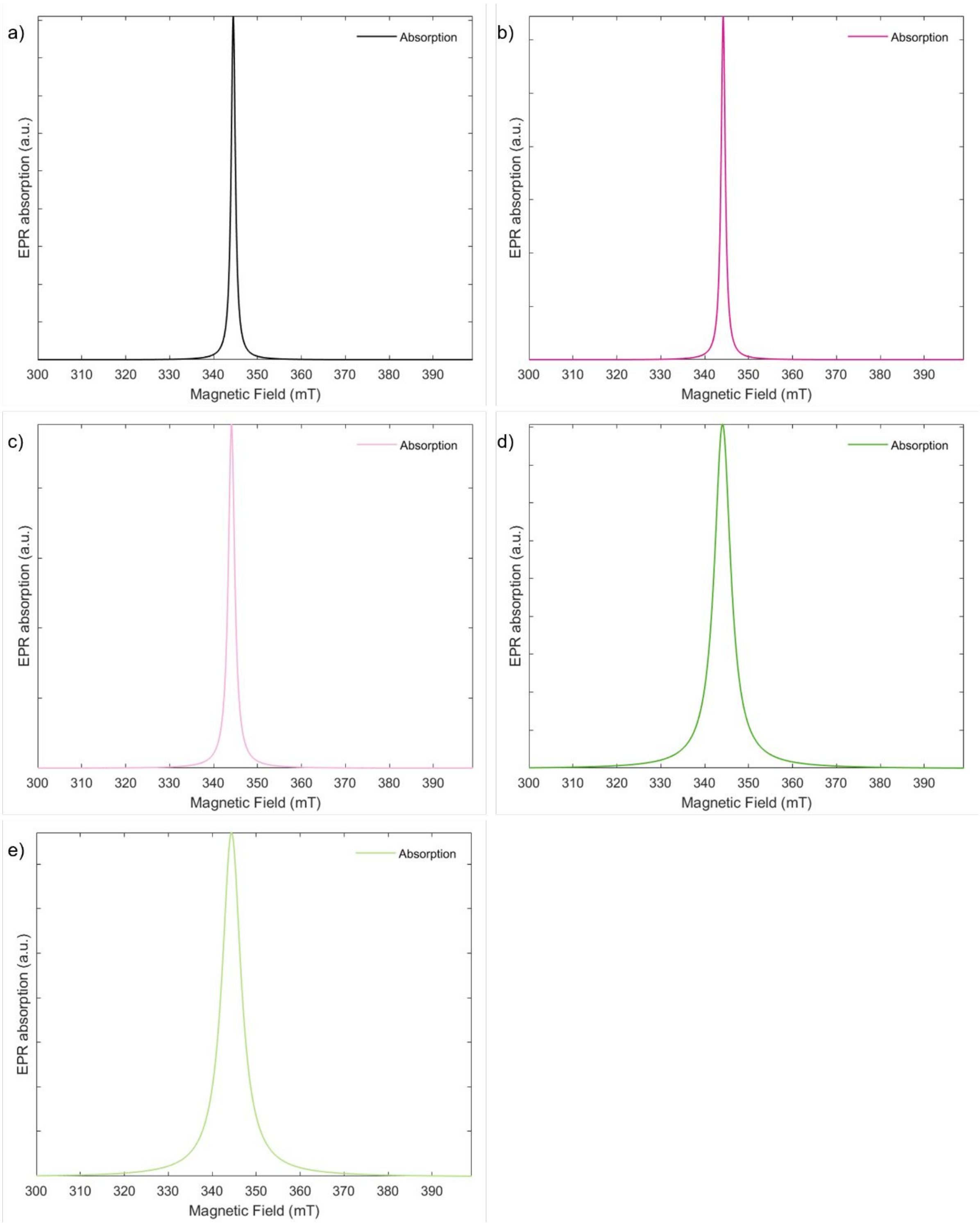

Figure S10. EPR absorption signal of a) pristine BN, b) P-BN_{IL-HF}, c) P-BN_{IL-LF}, d) P-BN_{PA-HF}, and e) P-BN_{PA-LF}, as derived from the integration of simulated EPR signals from Fig.S9. By integrating the absorption signals, we obtained the concentration of -OB_3 species existing in our samples, responsible for the EPR signal.

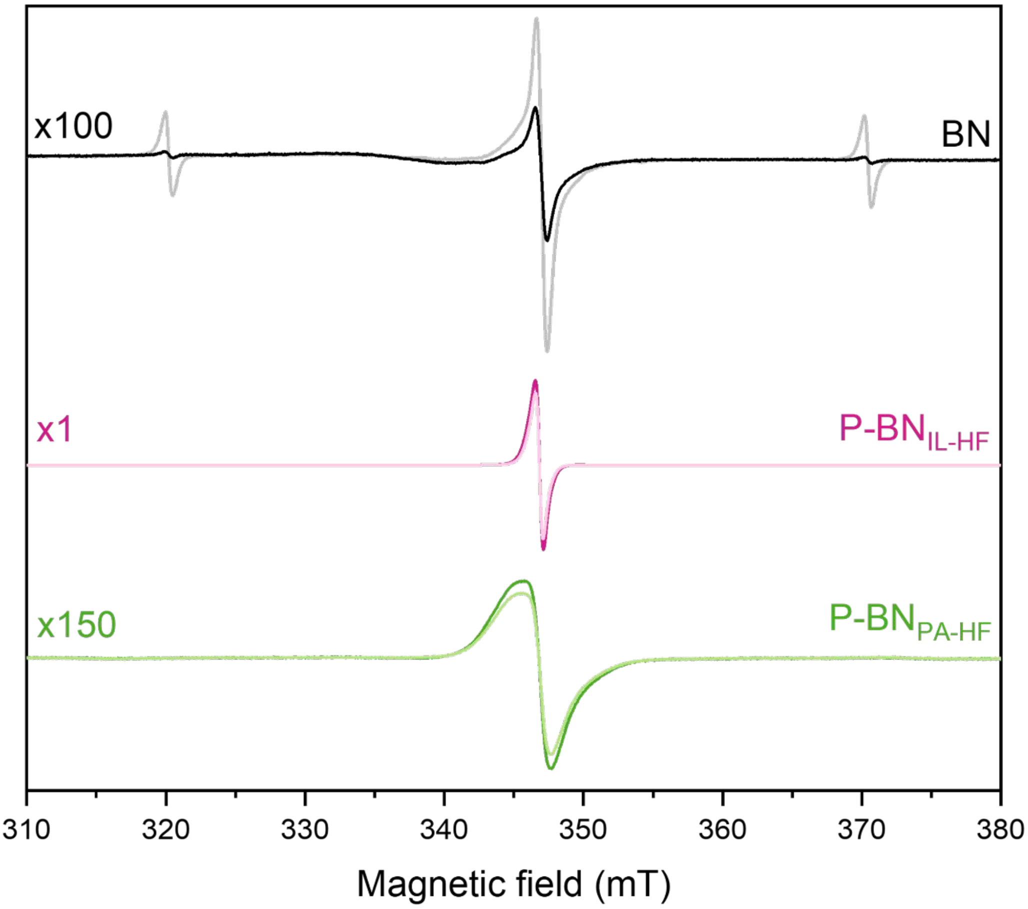

Figure S11. EPR spectra of pristine BN and P-doped BN samples at -268 °C and He atmosphere. Multiplication factors are shown on the left side of the signals to facilitate comparison between samples. The spectra indicate change in signal before (darker colour) and after (lighter colour) irradiation with UV light source.

Table S4. Photocatalytic CO_2 reduction results under different conditions and control tests on pristine BN and P-doped BN samples. Except for the control tests, all measurements were repeated three times and the standard deviation was calculated.

Sample	Conditions	Light	^{12}CO evolution rate (μmol g^{-1} h^{-1})	$^{12}CH_4$ evolution rate (μmol g^{-1} h^{-1})
**BN**	$^{12}CO_2/H_2$	UV/Vis	2.689 ±0.245	0.244 ±0.041
**BN**	$^{12}CO_2/H_2$	Visible (> 400 nm)	0.101 ±0.013	n.d.*
**P-BN$_{PA-LF}$**	$^{12}CO_2/H_2$	UV/Vis	1.056 ±0.170	0.135 ±0.008
**P-BN$_{PA-LF}$**	$^{12}CO_2/H_2$	Visible (> 400 nm)	0.019 ±0.003	n.d.
**P-BN$_{IL-LF}$**	$^{12}CO_2/H_2$	UV/Vis	0.543 ±0.032	0.016 ±0.001
**P-BN$_{IL-LF}$**	$^{12}CO_2/H_2$	Visible (> 400 nm)	0.054 ±0.016	n.d.
		**Control tests**		
**No catalyst**	$^{12}CO_2/H_2$	UV/Vis	n.d.	n.d.
**BN**	$^{12}CO_2/H_2$	No irradiation	n.d.	n.d.
**P-BN$_{PA-LF}$**	$^{12}CO_2/H_2$	No irradiation	n.d.	n.d.
**BN**	N_2/H_2	UV/Vis	2.370	0.489
**BN**	$^{13}CO_2/H_2$	UV/Vis	3.076	0.124
**P-BN$_{PA-LF}$**	$^{13}CO_2/H_2$	UV/Vis	0.796	0.085

*n.d. = not detected

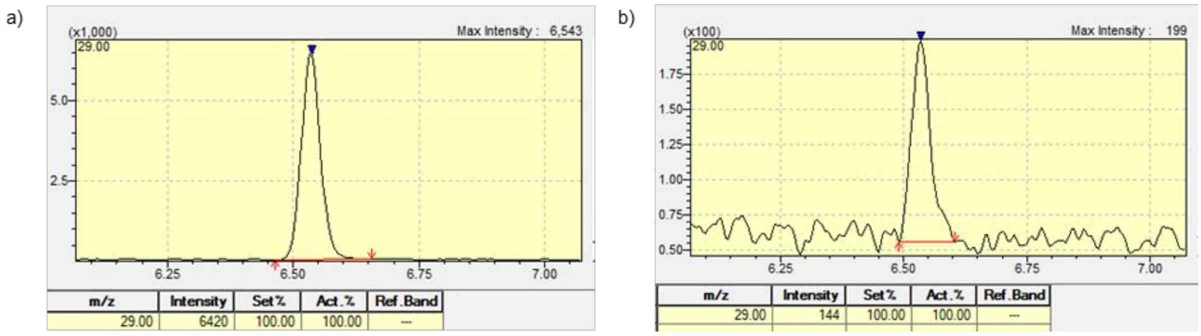

Figure S12. Confirmation of photocatalytic CO_2 conversion to CO. The ^{13}CO (m/z = 29) peak presented at a) for BN and b) for P-BN_{PA-LF} was formed after photocatalytic tests in gas phase using $^{13}CO_2/H_2$ flow. We note that the $^{13}CO_2$ cylinder also contains $^{12}CO_2$ traces, and it is inevitable that $^{12}CO_2$ and $^{13}CO_2$ initially coexist in our system. As a result, there is production of both ^{12}CO and ^{13}CO during irradiation. The produced ^{13}CO:^{12}CO ratio is – based on their respective peak intensity ratio – 0.2 for BN and 0.02 for P-BN_{PA-LF}.

References

(1) L'Hermitte, A.; Dawson, D. M.; Ferrer, P.; Roy, K.; Held, G.; Tian, T.; Ashbrook, S. E.; Petit, C. Formation Mechanism and Porosity Development in Porous Boron Nitride. *J. Phys. Chem. C* **2021**, *125* (49), 27429–27439. https://doi.org/10.1021/acs.jpcc.1c08565.

(2) Mistry, E. D. R.; Lubert-Perquel, D.; Nevjestic, I.; Mallia, G.; Ferrer, P.; Roy, K.; Held, G.; Tian, T.; Harrison, N. M.; Heutz, S.; et al. Paramagnetic States in Oxygen-Doped Boron Nitride Extend Light Harvesting and Photochemistry to the Deep Visible Region. *Chem. Mater.* **2023**, *35* (5), 1858–1867. https://doi.org/10.1021/acs.chemmater.2c01646.

(3) Tian, T.; Hou, J.; Ansari, H.; Xiong, Y.; L'Hermitte, A.; Danaci, D.; Pini, R.; Petit, C. Mechanically Stable Structured Porous Boron Nitride with High Volumetric Adsorption Capacity. *J. Mater. Chem. A* **2021**, *9* (22), 13366–13373. https://doi.org/10.1039/d1ta02001c.
